# Evaluation of anti-liver cancer activity and anticancer mechanism of one novel small molecule compound (THY-10A62) targeting FAK pathway

**DOI:** 10.3389/fonc.2025.1498005

**Published:** 2025-09-01

**Authors:** Wanqiu Huang, Rong Zou, Jie Xu, Yuliang Deng, Dongping Zhang, Yiguo Hu, Qian Zhang, Jian Huang, Zhaoqi Zhang

**Affiliations:** ^1^ Key Laboratory of Systems Biomedicine (Ministry of Education), Shanghai Centre for Systems Biomedicine, Shanghai Jiao Tong University, Shanghai, China; ^2^ Department of Medicinal Chemistry, School of Pharmacy, Fudan University, Shanghai, China; ^3^ Department of Thyroid Surgery and National Clinical Research Center for Geriatrics, State Key Laboratory of Biotherapy and Cancer Center, West China Hospital, Sichuan University, and Collaborative Innovation Center for Biotherapy, Chengdu, China; ^4^ Department of Breast and Thyroid Surgery, Shanghai General Hospital, Shanghai Jiao Tong University School of Medicine, Shanghai, China

**Keywords:** liver cancer, small molecule compound, THY-10A62, FAK inhibitor, mechanism, signal pathway

## Abstract

**Introduction:**

Focal adhesion kinase (FAK) is a promising therapeutic target, and its aberrant overexpression has been implicated in the growth and metastasis of multiple cancers, including hepatocellular carcinoma (HCC). This study preliminarily evaluated the antitumor activity and mechanisms of THY-10A62, a novel FAK inhibitor, *in vivo*.

**Methods:**

The maximum tolerated dose (MTD) and median lethal dose (LD_50_) of THY-10A62 were determined in ICR mice using dose-escalation survival and tolerability assessments. Antitumor efficacy was tested in an HCC-LM3 cell line-derived xenograft (CDX) model and in patient-derived xenograft (PDX) models of liver cancer. FAK pathway modulation was examined using a protein phosphorylation chip coupled with network pharmacology analyses. FAK phosphorylation levels were measured in PDX tumors following treatment. Efficacy was benchmarked against PF-562271.

**Results:**

The MTD of THY-10A62 in mice was < 45 mg/kg, and the LD₅₀ in female mice was 49 mg/kg. At 15 mg/kg, THY-10A62 significantly inhibited liver tumor growth (TGI > 40%), with greater suppression than PF-562271 at the tested conditions. In PDX tumors, THY-10A62 markedly down-regulated FAK phosphorylation. Phospho-proteomic profiling indicated altered phosphorylation of downstream effectors, including BRAF and RASGRF1, consistent with inhibition of FAK-mediated signaling.

**Discussion:**

THY-10A62 demonstrates *in vivo* antitumor activity against HCC with an acceptable tolerability window, supporting FAK as a viable target. The observed reductions in FAK phosphorylation and changes in BRAF and RASGRF1 phosphorylation suggest pathway-level modulation underlying efficacy. These findings provide preliminary evidence that THY-10A62 is a potential FAK inhibitor for liver cancer therapy and warrant further studies to refine dosing, characterize pharmacokinetics/toxicity, and validate efficacy across additional HCC models.

## 1 Introduction

Liver cancer is the third cause of cancer-related death worldwide (1). constituting the predominant pathological subtype, accounting for over 80% of cases. Hepatocellular carcinoma (HCC) accounts for 70 to 85% pathological subtype of liver cancers (2). Due to its insidious onset and rapid progress, HCC often diagnosed at a progressed stage or exhibiting distant metastasis, leading to a poor clinical outcome (3).

For a period of ten years, sorafenib was the exclusive approved first-line treatment for advanced HCC (4). Sorafenib exerts its anticancer activity by inhibiting tumor angiogenesis through signaling pathways involving vascular endothelial growth factor receptor (VEGFR), platelet-derived growth factor receptor (PDGFR) (5) and Raf/MEK/ERK signaling cascade (6). However, sorafenib treatment can lead to off-target effects and adverse reactions. Moreover, the objective response rate of sorafenib remains relatively low, at only 2-3% (7, 8). Most patients develop resistance within 6–9 months (9). Therefore, pursuing novel therapeutic agents for HCC holds paramount clinical significance.

Focal adhesion kinase (FAK) is a tyrosine kinase (10). Compelling evidence suggests that FAK is overexpressed in various types of tumors, including lung cancer (11), breast cancer (12), gastric cancer (13). Our preliminary research has demonstrated a significant upregulation of FAK in liver cancer tissues (14). Fujii reported FAK mRNA overexpression in HCC cells compared to corresponding non-cancerous liver tissue (15). Researches showed that FAK was correlated with the aggressiveness of HCC (16) and was associated with significantly poorer survival (17). FAK can also activate downstream signaling pathways involved in cell proliferation, differentiation, invasion, and metastasis (18, 19). FAK would be a promising target for HCC therapeutics. Currently, several small-molecule inhibitors targeting FAK phosphorylation level have been reported to exhibit antitumor effects such as Conteltinib (20), PF-562271 (21), GSK-2256098 (22), Defactinib (23), and PND-1186 (24). Although several FAK inhibitors, such as Defactinib, have shown promise and are undergoing clinical evaluation, including receiving FDA Fast Track designation for combination therapy in mesothelioma (25), no FAK-targeting small molecule has yet received full regulatory approval for standard clinical use. Most FAK inhibitors remain in preclinical development or early-phase (Phase I/II) clinical trials (26).

In our previous research, we employed a pharmacophore-based screening strategy to design and synthesize a class of FAK inhibitors with a 5-fluoro-7H-pyrrolo[2,3-d] pyrimidine scaffold (27). Among these, 2-((2-((3-(Acetamidomethyl)phenyl)amino)-5-fluoro-7H-pyrrolo[2,3-d]pyrimidin-4-yl)amino)-N-methylbenzamide (THY-10A62 or 16c) displayed an IC50 value of 12 nM against FAK kinase activity and half-maximal inhibitory concentrations of 2.39 μM and 10.07 μM against the liver cancer cell lines YY8103 and SMMC7721, respectively (27). Through intraperitoneal administration, THY-10A62 demonstrated superior *in vivo* anti-tumor proliferation activity compared to PF-562271 at equivalent dosages in a SMMC7721 subcutaneous xenograft mouse model. Building upon our preliminary research, we utilized NSG mice to establish a PDX subcutaneous liver cancer tumor model. This allowed us to comprehensively evaluate the anticancer efficacy of THY-10A62, its tissue distribution, potential FAK targets and signaling pathways, as well as its association with clinical prognosis in liver cancer. Our objective is to gain a comprehensive understanding of how THY-10A62 inhibits liver cancer cell proliferation through modulation of the FAK signaling pathway, providing a solid foundation for further development of liver cancer therapeutics.

## 2 Materials and methods

### 2.1 Chemical synthesis of THY-10A62

THY-10A62 was synthesized as previously described over 5 steps with an overall yield of 4.5% (34). The detailed synthetic route is depicted in **Supplementary Figure 1**.

### 2.2 Detection of MTD and LD_50_ of THY-10A62 in ICR mice

In this study, 115 five-week-old ICR (Institute of Cancer Research) mice were purchased as test mice, consisting of 65 females and 50 males. Preliminary experiments were conducted to determine the appropriate dosage of the test substance, followed by the formal experimental groupings. Male and female mice were divided into nine groups of three mice each. The test substances were administered via tail vein injection with different doses. Test mice were closely observed continuously twice daily from the day of administration until 14 days post-dose, and detailed records were kept to document any toxic reactions or deaths. The weight of test mice was measured before administration (D0) and after (D3, D7, D10, D14) the administration of test substances. The MTD of THY-10A62 were calculated based on observed mortality of ICR mice. MTD should be smaller than 45mg/kg. The LD50 of THY-10A62 was calculated using Bliss algorithm and SPSS19 software. Tissues were preserved in 10% paraformaldehyde for subsequent pathological analysis.

### 2.3 Construction of liver cancer tissue CDX model mice

A total of 64 female Balb/c-Nude mice were selected for the study. Four nude mice were inoculated with LM3 cells to establish donor tumors, each receiving an inoculation of 1×10^7^ cells (100 ul). Once the tumors reached a size of 400–600 mm^3^, the mice were euthanized by CO_2_ asphyxiation and the tumors were excised and cut into 2 mm×2 mm fragments. These tumor fragments were then transplanted into the right shoulder and back of the remaining 60 nude mice using a vaccination needle. When the average tumor volume reached 100mm^3^, group testing was conducted. The experiment consisted of six groups with six animals in each group: Vehicle control, THY-10A62 at doses of 5mg/kg, THY-10A62 at doses of 10 mg/kg, THY-10A62 at doses of 15mg/kg, Sorafenib at a dose of 15mg/kg, and PF-562271 at a dose of 15mg/kg. The drugs were administered every three days and tumor volume and weight measurements were taken simultaneously. After completion of the experiment, tumors were harvested for weighing purposes and photographs were collected and processed. Evaluation indices included relative tumor inhibition rate and tumor growth inhibition rate.

### 2.4 Construction of liver cancer tissue PDX model mice

SPF grade NSG mice (Shanghai Southern Model Biological Technology Co., Ltd., Production License Number: SCXK (Hu) 2017-0010, Certificate Number: 20170010024094) was used to construct the PDX model of HCC tissues. 5-week-old mice weighing 16-17g were purchased. During the formal experiments, the test mice were 6 weeks old weighing 18-20g. All mice that did not meet the quality requirements were excluded from this study. The origin tumor tissue from a 58-year-old male with positive HBV was designed as eBPDX-001. The tumor tissue from a 73-year-old male patient with HBV positive was designed as eBPDX-002. The tumor tissue from a 63-year-old male with HBV negative was designed as eBPDX-003. eBPDX-001 and eBPDX-003 served primarily as validation models for tumorigenicity during the early experimental stages. eBPDX-002-derived PDX model as the representative *in vivo* model for efficacy assessment of THY-10A62. Tumor tissues were placed in physiological saline on ice and were dissected into uniform fragments of approximately 2cm x 2cm in size, separately. Then the tumor fragments were immediately implanted subcutaneously on the shoulders of female NSG mice. After implantation, tumor size and weight were observed and recorded daily. The tumor tissue from tumorigenic mice were designated as F1-PDX. When the F1-PDX xenograft tumors reached approximately 5mm x 5mm in dimension or 400–500 mm^3^ in size, the mice were euthanized by CO_2_ asphyxiation and the tumor tissue was obtained. The same procedure was repeated to obtain F2-PDX tumor tissue. When the volume of F2-PDX xenograft tumors reached approximately 100 mm^3^ in female NSG mice, follow-up group tests were conducted.

### 2.5 Ethics approval and consent to participate

Animal experiments in this study were conducted in full compliance with the ARRIVE guidelines on the protection of animals used for scientific purposes. All procedures were carried out in accordance with these internationally recognized ethical standards and regulations. All experimental procedures involving animals were reviewed and approved by the Experimental Animal Ethics Committee of Chengdu Yibang Pharmaceutical Technology Co., Ltd. (Approval No.: 202101052).

For the preparation of mouse PDX models using human tumor samples, ethical approval for the collection and use of human tissue was obtained from the Biomedical Ethics Committee of West China Hospital, Sichuan University [Approval No.: HX2019 (905)]. Informed consent was obtained from all individuals who provided tumor samples for this study, and their privacy and personal information were rigorously protected in line with ethical guidelines. All research involving human tissue was conducted in full compliance with the principles set forth in the Declaration of Helsinki.

### 2.6 Main experimental drugs preparation

Solvent Preparation: Add DMSO (Catalogue ID SHBM5161, Sigma-Aldrich), Kollipor^®^ HS 15 (Catalogue ID 70142-34-6, Sigma-Aldrich), and saline into the tubes. Then vortex these components to mix thoroughly. The final concentration of DMSO was 5%, solutol 10%, and saline 85%. Store these solutions in a place protected from light at 4°C and use within 72 hours. Mass spectrometry was used to test the stability of experimental drugs. The same solvent composition was used for the control group to rule out potential confounding effects of the solvent components.

THY-10A62 Solution Preparation: The required amount of THY-10A62 (Home-made) was weighed and added into EP tube, then slowly add DMSO (Catalogue ID SHBM5161, Sigma-Aldrich) until complete dissolution. Next, add solutol and vortex to mix thoroughly. Finally, add physiological saline and mix thoroughly until no crystal precipitated. The final concentration of DMSO was 5%, solutol 10%, and saline 85%. Store these solutions in a place protected from light at 4°C and use within 72 hours. Mass spectrometry was used to test the stability of experimental drugs.

PF-562271 Solution Preparation: The required amount of PF-562271 (Catalogue ID S289004, Selleckchem) was weighed and added into EP tube, then slowly add DMSO solution (Catalogue ID SHBM5161, Sigma-Aldrich) until complete dissolution. Then add PEG400 and vortex to mix thoroughly, followed by the addition of Tween 80 and vortex thoroughly. Finally, add purified water to achieve the desired concentration, resulting in the final concentration of DMSO was 5%, PEG400 40%, Tween-80 5%, and purified water 50%. Store these solutions in a place protected from light at 4°C and use within 72 hours. Mass spectrometry was used to test the stability of experimental drugs.

The “S-type” grouping method was used in this study, with four experimental groups: Vehicle, THY-10A62 (5 mg/kg), THY-10A62 (15 mg/kg), and PF-562271 (15 mg/kg). Each group consisted of 6 female mice. The test mice were administered via tail vein injection on the same day as grouping. Subsequent administrations were given every 3 days, with a dosage of 10 ml/kg per administration. In total, this study involved six administrations.

### 2.7 Observation records of experimental animal

All surviving experimental animals were observed for a minimum of two weeks throughout the experiments. Observations encompassed general appearance, behavioral status, eyes, oral cavity, nasal and oral areas, ears, fur condition, feces, urine, and reproductive organs and other signs of toxicity. In addition, the body weight of the mice was measured every 3 days. At the end of the drug treatment period, tumor tissues were collected from each group of experimental animals after euthanasia with an approved method.

The tumor weights were measured and used to calculate the Tumor Growth Inhibition Rate (IR_TW_%). Additionally, photographs of the tumor appearance were taken. IR_TW_% = [(Mean Tumor Weight in Control Group - Mean Tumor Weight in Treatment Group)/Mean Tumor Weight in Control Group] x 100%. IR_TW_% < 40% was considered ineffective, while IR_TW_% ≥ 40% and statistical significance (p < 0.05) was considered effective. During the drug treatment period, the long diameter (a) and short diameter (b) of the tumor tissue were measured using calipers. Tumor Volume = 0.5 × a × b². Relative Tumor Growth Rate, T/C (%) = (TRTV/CRTV) × 100%, where TRTV represents the Relative Tumor Volume of the treatment group, and CRTV represents the Relative Tumor Volume of the negative control group. RTV (Relative Tumor Volume) = Vt (tumor volume at each measurement)/V_0_ (tumor volume at the start of dosing, i.e., D0). Tumor Growth Inhibition (TGI) =1-T/C. TGI < 40% was considered ineffective, while TGI% ≥ 40% with statistical significance (p < 0.05) was considered effective.

### 2.8 Tissue distribution analysis

On the last day of the experiment, two hours after the final administration, major organs from the mice were collected, and homogenates of the heart, liver, spleen, lung, kidney, and tumor tissues were prepared. The detection was performed using an analytical method established on the AB SCIEX Triple Quad™ 5500+ mass spectrometer for PF-562271 and THY-10A62. Standard curves were prepared at concentrations of 10, 30, 100, 300, 1000, and 3000 ng/mL, and data were collected using Analyst software and analyzed with Sciex OS software.

The liquid chromatography conditions were as follows: Flow rate: 0.3 mL/min, Sample chamber temperature: 10°C, Column temperature: 40°C, Injection volume: 3 µL, Column: ACQUITY UPLC^®^ BEH C18 1.7μm, 2.1×100mm column. The mass spectrometry parameters were configured as follows: Electrospray ionization (ESI) in positive ion mode, Capillary voltage: 5.5 kV, Ion source temperature: 500°C, Declustering potential (DP): 170 V, Multiple Reaction Monitoring (MRM) mode was used to detect PF-562271, THY-10A62, and the internal standard SAHA. These analytical methods were employed to accurately determine the concentrations of PF-562271 and THY-10A62 in the tissue homogenates for further analysis.

### 2.9 Immunohistochemical analysis

The epithelial cell adhesion molecule (EpCAM) is expressed in both hepatic progenitor cells and hepatocellular carcinoma (HCC), and is considered a marker for liver cancer stem cells. To assess the degree of tumor infiltration in different groups, we examined the expression levels of EpCAM in liver and lung tissues based on tissue distribution results. The expression level of EpCAM may reflect the extent and nature of tumor cells within these tissues.

Follow the instructions of the immunohistochemical kit (D601037-0050) provided by Sangon Biotech (Shanghai) Co., Ltd., the tissue sections were soaked with xylene for deparaffinization, then sequentially hydrated with 95%, 85%, and 75% ethanol. The sections were rinsed three times with distilled water (ddH_2_O). A 10 mmol/L citrate buffer (pH 6.0) solution was used to perform antigen retrieval. Again washing, enzyme inactivation, and rinsing, the sections were blocked with PBST containing 3% BSA. The primary antibody was applied in a humid chamber for overnight incubation. The next day, the sections were warmed to room temperature, washed and incubated with an enzyme-labeled secondary antibody. Color was developed by DAB method. The reaction was stopped at the appropriate time by rinsing with distilled water. The sections were counterstained with hematoxylin, followed by differentiation using hydrochloric acid-ethanol and returned to blue using PBST. Finally, dehydrate the sections by 75%, 85%, 95% ethanol in sequence, and replaced with xylene, then neutral resin was used to seal. The slides were then observed and photographed under a microscope.

### 2.10 Phosphoprotein profiling by phospho explorer antibody microarray

The Phospho Explorer antibody microarray (Full Moon Biosystems, Inc., Sunnyvale, CA) contains 304 antibodies. Each of the antibodies has two replicates that are printed on coated glass microscope slide, along with multiple positive and negative controls. The antibody array experiment was performed by Wayen Biotechnology (Shanghai, China) according to their established protocol. In brief, cell lysates obtained from NC and Tumor were biotinylated with Antibody Array Assay Kit (Full Moon Biosystems, Inc.). The antibody microarray slides were first blocked in a blocking solution (Full Moon Biosystems, Inc) for 30 min at room temperature, rinsed with Milli-Q grade water for 3–5 min, and dried with compressed nitrogen. The slides were then incubated with the biotin-labeled cell lysates (~ 100 μg protein) in coupling solution (Full Moon Biosystems, Inc.) at room temperature for 2 h. The array slides were washed 4–5 times with 1X Wash Solution (Full Moon Biosystems, Inc.) and rinsed extensively with Milli-Q grade water before detection of bound biotinylated proteins using Cy3-conjugated streptavidin. The slides were scanned on a GenePix 4000 scanner and the images were analyzed with GenePix Pro 6.0 (Molecular Devices, Sunnyvale, CA). The fluorescence signal (I) of each antibody was obtained from the fluorescence intensity of this antibody spot. The data was normalized by the total fluorescence intensity. A ratio computation was used to measure the extent of protein phosphorylation and protein expression respectively. Foldchange >1.20 or foldchange <0.83 were considered as differential expression.

### 2.11 Quantitative real-time PCR

Total RNA was extracted from mouse tumor tissues using the standard TRIzol reagent method (Invitrogen, USA) according to the manufacturer’s protocol. Reverse transcription was performed using the PrimeScript™ FAST RT Reagent Kit with gDNA Eraser (Takara, Japan) to eliminate genomic DNA contamination and synthesize first-strand cDNA, following the supplier’s instructions.

qPCR was carried out using the TB Green^®^ Premix Ex Taq™ (Tli RNaseH Plus) (Takara, Japan) on a Roche LightCycler^®^ 96 System. The thermal cycling conditions were as follows: initial denaturation at 95°C for 30 seconds, followed by 40 cycles of denaturation at 95°C for 5 seconds and annealing/extension at 60°C for 30 seconds. Melting curve analysis was performed using a three-step protocol: 95°C for 5 seconds, 60°C for 60 seconds, followed by a gradual increase to 95°C to generate the dissociation curve.

Gene-specific primers were designed for the following target genes: PTK2 (FAK), BRAF, BAD, AKT, and RASGRF1. All primers were synthesized by Sangon Biotech Co., Ltd. (Shanghai, China). All reactions were performed in triplicate. Relative mRNA expression levels were calculated using the 2^-ΔΔCt method, with normalization to the internal control gene GAPDH.

### 2.12 Protein network relationship analysis using network pharmacology method

The GeneCards database (https://www.genecards.org) was searched with “hepatocellular carcinoma” and “HCC” as the key words to obtain the results of disease-related targets. In order to facilitate the subsequent shared gene analysis, we set relevance score >=10 as the screening threshold, and 1602 target genes were obtained.

We used the differentially expressed proteins in the Phospho Explorer Antibody Microarray detection results as potential targets of THY-10A62, then we matched the protein names to gene names in the Uniprot database (https://www.uniprot.org/). The differentially expressed genes obtained by phosphorylation microarray and the HCC-related targets obtained from the database search were imported into the jvenn online mapping software (https://jvenn.toulouse.inrae.fr/) to do the intersection analysis, and then the Venn diagram was drawn, and the intersection target was considered as the key target of THY-10A62 for the treatment of HCC. We uploaded the intersected key target genes to Metascape (https://metascape.org), set the species to Homo sapiens, and performed GO and KEGG enrichment with a significance threshold of P < 0.01; the results were downloaded for further analysis.

The confidence level of STITCH online tool (http://stitch.embl.de/) was set to be greater than 0.4, and the chemical components without relevant information were excluded. Cytoscape 3.9.1 was employed to construct the “THY-10A62-HCC-arget gene-pathway” PPI network, and network topology parameters were analyzed, with nodes ranked by degree value to verify THY-10A62 and its core targets within the HCC pathway.

The core targets obtained above were subjected to molecular docking validation. The 3D structures of the small molecules were downloaded from PubChem database, and the crystal structures of the core target proteins used for docking were downloaded from PDB database. All the proteins were processed by PyMol 2.5 software, including the removal of water molecules, salt ions and small molecules. AutoDock Vina 1.1.2 software was used for molecular docking and PyMol 2.5 was used to visualize the docking results.

### 2.13 Statistical analysis

All data are presented as mean ± SEM. Statistical analyses were performed using Excel 2021, SPSS 19.0, and GraphPad Prism 9. Homogeneity of variance was assessed with Levene’s test. Data with equal variances (p > 0.05) were analyzed by one-way ANOVA followed by Dunnett’s t-test for multiple comparisons, whereas data with unequal variances (p < 0.05) were analyzed using the Kruskal–Wallis H test followed, if significant, by the Mann–Whitney U test. A p-value < 0.05 was considered statistically significant.

### 2.14 AI usage statement

To improve clarity and readability, we used ChatGPT (OpenAI; model: GPT-4; accessed August 2025) for language editing. The AI outputs were used only as drafts; all content was fact-checked, critically revised, and finalized by the authors.The tool had no role in study design, data collection, statistical analysis, or interpretation of results.

## 3 Results

### 3.1 Analysis of MTD and LD_50_ of THY-10A62 in ICR mice

**Figure 1:**
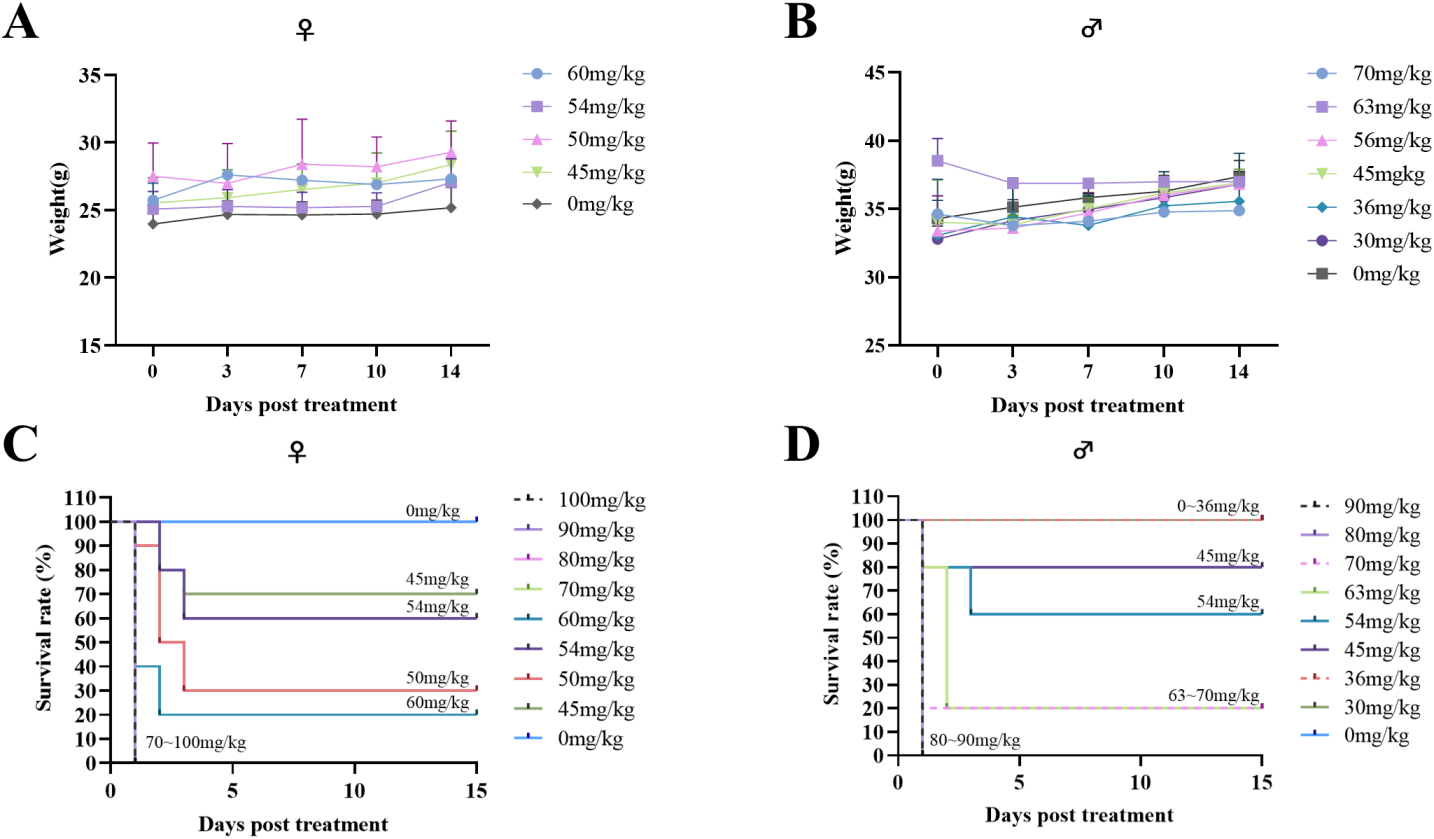
Acute tolerance to THY-10A62 toxicity in ICR mice. Following intravenous administration of varying concentrations of THY-10A62, temporal changes in body weight are shown for female **(A)** and male **(B)** mice, and survival curves are presented for female **(C)** and male **(D)** mice. Each dose group initially included 7 mice. Data are expressed as mean ± SEM. Statistical comparisons were performed between each treatment group and the vehicle group at corresponding time points. Error bars are not shown for some groups due to early mortality and insufficient surviving animals for valid calculation.

To detect the maximum tolerated dose (MTD) and median lethal dose (LD_50_) of THY-10A62 in mice, a stratified experimental design was implemented, segregating the test subjects into nine distinct groups, with a minimum of three individuals per group, for both genders. Post- administration, the test mice experienced transient body weight loss, which subsequently recovered over time (**Figures 1A, B**). The primary body weight reduction was observed within the initial three days after administration (**Supplementary Tables 1**, **2**).

In the female cohort, the control group demonstrated a weight change rate of 2.92% (**Supplementary Table 1**). In the treatment groups, all subjects in the four highest dosage groups (100 mg/kg, 90 mg/kg, 80 mg/kg, 70 mg/kg) died of the treatment on the day of administration, precluding measurement of their weight changes. Among the remaining groups (60 mg/kg, 54 mg/kg, 50 mg/kg, 45 mg/kg), the weight change rates varied from -0.26% to 4.21% (**Supplementary Table 1**).

In the male cohort, the control group exhibited a weight change rate of 2.42% (**Supplementary Table 2**). In the treatment groups, all mice in the two highest dosage groups (90 mg/kg, 80 mg/kg) perished on the day of administration and their weights could not be measured. The weight change rates of the remaining groups (80 mg/kg, 70 mg/kg, 60 mg/kg, 54 mg/kg, 50 mg/kg, 45 mg/kg) ranging from -4.26% to 4.18% (**Supplementary Table 2**).

Furthermore, the MTD for THY-10A62 was determined to be below 45 mg/kg for both male and female mice (**Figures 1C, D**). The LD_50_ was determined to be 49 mg/kg for the female mice (**Supplementary Table 3**) and 57 mg/kg for the male mice (**Supplementary Table 4**). Notably, no significant changes in body weights were observed among the surviving subjects, with no statistically differences. The higher LD_50_ observed in male mice relative to the female mice indicates a greater tolerance of male mice towards THY-10A62. Therefore, female mice were selected as experimental objects for the subsequent efficacy experiments.

### 3.2 THY-10A62 effectively inhibited the growth of liver cancer in HCC-LM3 CDX mice

**Figure 2:**
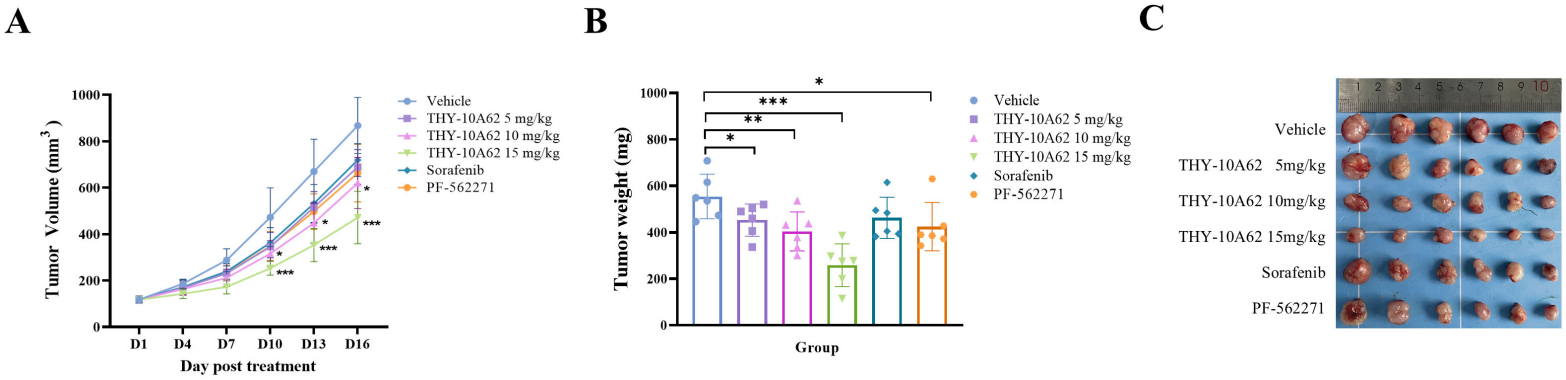
The inhibitory effects of THY-10A62, Sorafenib and PF-562271 on the HCC-LM3 CDX tumor in Balb/c-Nude mice. Following intravenous administration of varying drug concentrations, **(A)** the temporal change curve of tumor volume was plotted; **(B)** the temporal change curve of tumor weight was generated; **(C)** the image depicting the physical appearance of excised tumor masses from CDX mice captured sequentially was presented. *p < 0.05; **p < 0.01; ***p < 0.001 v.s. Vehicle group.

To evaluate the inhibitory effect of THY-10A62 on HCC progression, we evaluated its impact on subcutaneous tumors in an HCC-LM3 cell line-derived xenograft (CDX) mouse model through intravenous administration via the tail vein. The CDX model was established using nude mice, and the study was divided into six treatment groups based on our previous experimental results: a Vehicle control group, and THY-10A62 treatment groups at doses of 5mg/kg, 10 mg/kg and 15mg/kg, alongside Sorafenib at doses of 15mg/kg and PF-562271 at 15mg/kg. The treatments were administered every three days over a 16-day period with tumor volume measurements taken at regular intervals (**Figure 2A**). Upon termination of the study on Day 16, the subcutaneous tumors were excised and their weights were documented (**Figures 2B, C**, **Supplementary Table 5**). The data revealed that THY-10A62, at a dose of 15mg/kg, exhibited superior antitumor activity compared to Sorafenib and PF-562271.

The relative tumor growth inhibition rate (TGI%) and the inhibition rate of tumor growth (IR_TW_%) were calculated to quantify the therapeutic effects. The results demonstrated that he TGI % of each treatment group, including THY-10A62 at 5mg/kg, 10 mg/kg, and 15mg/kg, Sorafenib at 15mg/kg, and PF-562271 at 15mg/kg, were found to be 20.5%, 28.6%, 45.6%, 17.7% and 24.2%, respectively (**Table 1**). Notably, the TGI % for the THY-10A62 (15 mg/kg) exceeded 40%, achieving statistical significance when compared to the Vehicle group (P<0.001) (**Table 1**). Furthermore, the IR_TW_% for THY-10A62–5 mg/kg, THY-10A62–10 mg/kg, THY-10A62–15 mg/kg, Sorafenib 15 mg/kg, and PF-562271–15 mg/kg were 26.7%, 27.2%, 53.4%, 16.6%, and 23.4%, respectively (**Table 2**). The IR_TW_% for THY-10A62–15 mg/kg was also exceeded 40% (**Table 2**). These findings indicate that THY-10A62, at a dosage of 15 mg/kg, exert a pronounced inhibitory effect on the growth of HCC-LM3 subcutaneous graft tumors, which indicated its potential as a novel therapeutic agent for HCC.

**Table 1:** The statistics of TGI (%) in CDX mice post-treatment.

Treatment	D3	D7	D10	D13	D16
Vehicle	0	0	0	0	0
THY-10A62–5 mg/kg	9.4	19.6	26.8	23.0	20.5
THY-10A62–10 mg/kg	12.4	26.6	33.3	33.3	28.6
THY-10A62–15 mg/kg	23.2	40.0	46.4^*^	47.3^*^	45.6^*^
Sorafinib	8.2	17.3	23.8	21.1	17.7
PF-562271	9.5	19.7	26.9	26.4	24.2

The day of group administration is D1. *p < 0.05 *vs*. Vehicle group.

**Table 2:** The statistics of tumor weight (mg) and IR_TW_% in CDX mice post-treatment.

Treatment	Tumor weight	IR_TW_%	Relative tumor weight (%)
Vehicle	554.6 ± 95.9		100
THY-10A62–5 mg/kg	452.9 ± 69.5^*^	26.7	81.7
THY-10A62–10 mg/kg	403.8 ± 84.4^**^	27.2	72.8
THY-10A62–15 mg/kg	258.5 ± 92.1^***^	53.4	46.6
Sorafinib	462.6 ± 88.7	16.6	83.4
PF-562271	424.8 ± 104.4^*^	23.4	76.6

Data are presented as mean ± SEM. *p < 0.05; **p < 0.01; ***p < 0.001 *vs*. Vehicle group.

### 3.3 THY-10A62 effectively inhibited the growth of liver cancer in PDX mice

To further evaluate the therapeutic potential of different doses of THY-10A62 and PF-562271 in the treatment HCC, we selected the eBPDX-002-derived PDX model, which was established from HBV-positive HCC tissue, as the representative *in vivo* model for efficacy assessment of THY-10A62.The study subjects consisting of six female mice per group, were randomly assigned into four treatment groups: Vehicle, THY-10A62 (5 mg/kg), THY-10A62 (15 mg/kg), and PF-562271 (15 mg/kg). The drug administration was initiated on the day of group assignment, followed by additional doses every three days for a total of five administrations over a 13-day observation period. All the test mice-maintained survival were observed without any abnormalities in their general condition, behavior, or physiological parameters of eyes, oral cavity, nasal region, ears, fur, feces, urine and reproductive organs.

**Figure 3:**
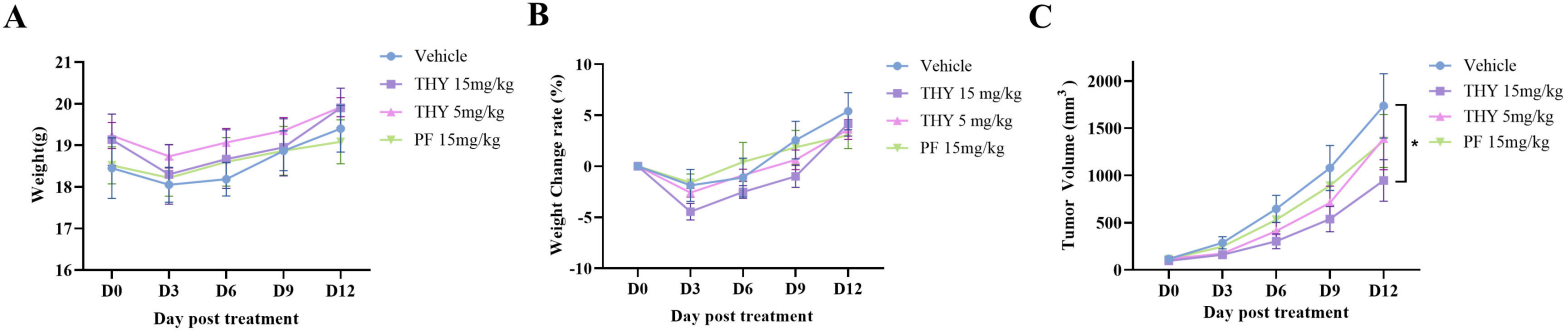
Acute tolerance to THY-10A62 toxicity and the inhibitory effects of THY-10A62 in PDX mice. **(A)** the temporal change curve of body weight over time; **(B)** the temporal change rate of body weight; **(C)** the temporal change curve of tumor volume change. *p < 0.05 v.s. Vehicle group.

Furthermore, the mice in the Vehicle group exhibited a stable increase in body weight, with an average weight gain of 5.40% by the end of the trial (**Figures 3A, B**, **Supplementary Tables 6**, **7**). In contrast, the mice in the THY-10A62 groups displayed varied weight gain at the end of the study, with increases of 3.61% for dose at 5 mg/kg, 4.16% for dose at 15 mg/kg, and 3.06% for PF-562271–15 mg/kg (**Figure 3A, B**, **Supplementary Tables 6**, **7**). There were no statistically significant differences observed in weight changes (p > 0.05).

At the end of 13-day observation, subcutaneous tumor growth in the THY-10A62 (15 mg/kg) group was significantly inhibited compared with the Vehicle group (p < 0.05). Tumor growth was also moderately suppressed in the THY-10A62 (5 mg/kg) and PF-562271 (15 mg/kg) treatment groups with no statistical significance (p > 0.05) (**Figure 3C**, **Supplementary Table 8**). These results suggest that THY-10A62 (15mg/kg) group exerts a substantial inhibitory effect on subcutaneous transplantation tumors derived from liver cancer PDX.

### 3.4 THY-10A62 modulates EpCAM expression in liver and lung tissues of PDX mice

**Figure 4:**
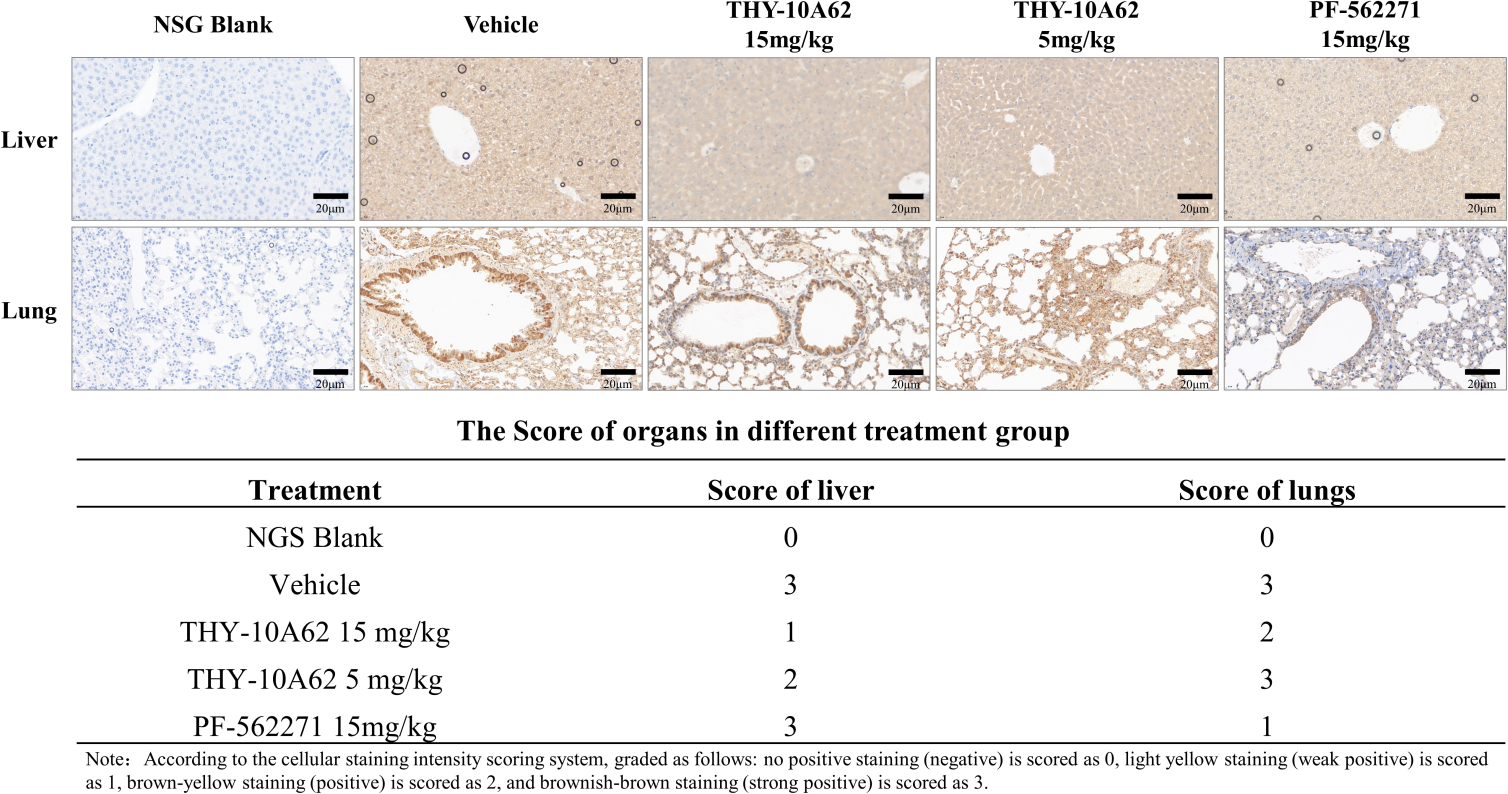
Immunohistochemical staining of EpCAM in liver and lung tissues from PDX mice treated with PF-562271 or THY-10A62. (Bar=20μm, 400×).

In our previously investigations, hematoxylin and eosin (HE) staining were used to assess tumor cell infiltration within the liver and lung tissues of PDX mice. EpCAM is known to be expressed in hepatic progenitor cells and HCC, and its overexpression has been reported to correlate with epithelial tumor burden in certain contexts (28). IHC results revealed elevated EpCAM expression in both liver and lung tissues of the Vehicle group, indicating the extensive tumor infiltration. EpCAM expression was decreased in lung tissue but remained elevated in the liver, suggesting a tissue-selective effect on tumor cell presence. (**Figure 4**). Conversely, in the THY-10A62 15mg/kg group, EpCAM expression was reduced in both liver and lung tissues, indicating a broader suppression of epithelial tumor cell presence (**Figure 4**). Although EpCAM is not a direct marker of metastasis, its expression pattern here may reflect the relative extent of tumor infiltration across different treatment groups. A weaker reduction was observed with THY-10A62 at 5 mg/kg, which may be related to differences in compound distribution (**Supplementary Figure 2**).

### 3.5 Potential molecular mechanism of inhibition of HCC proliferation by THY-10A62

**Figure 5:**
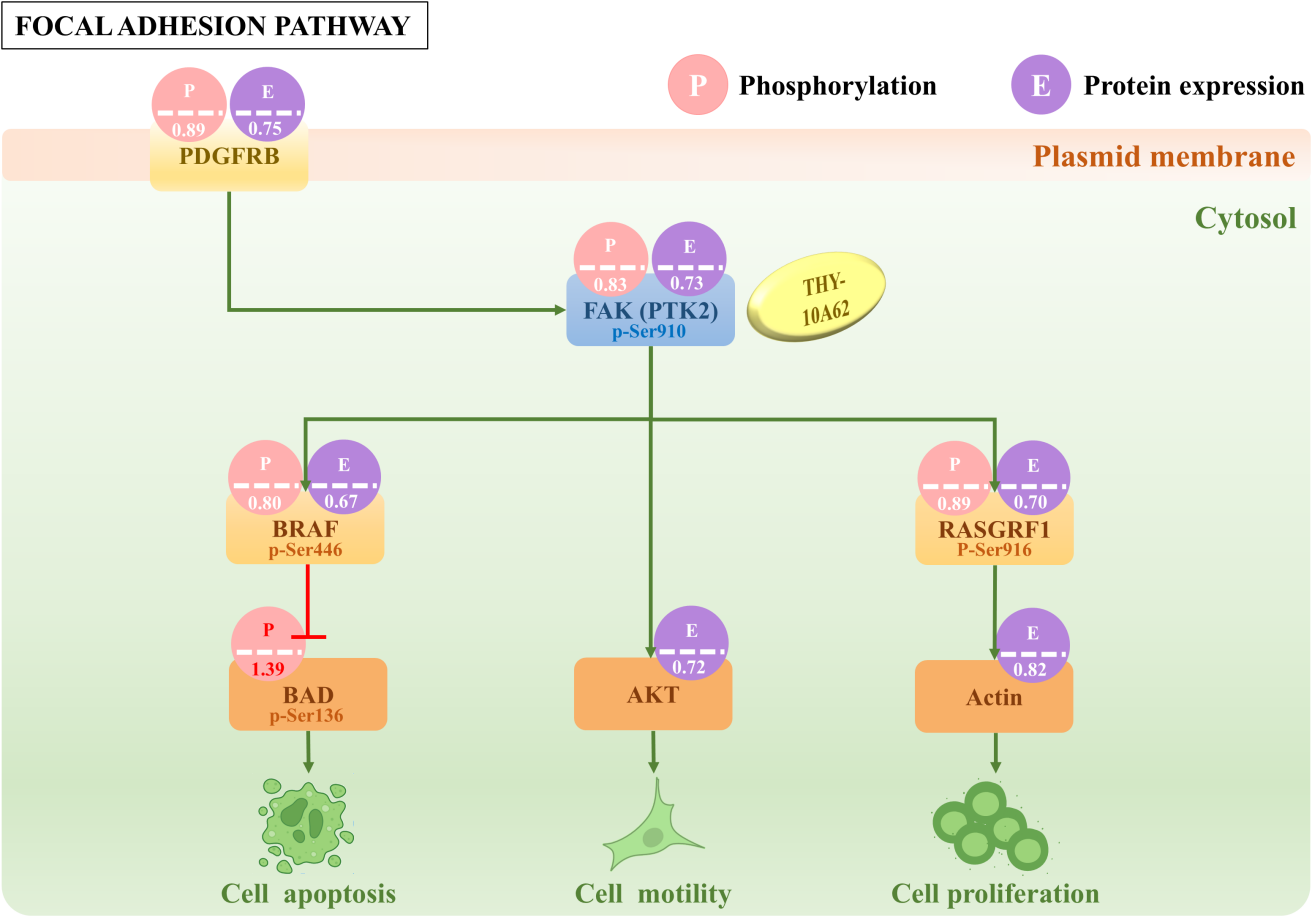
The difference of partial protein phosphorylation and protein expression in Focal adhesion signal pathway. The number in the picture indicates the foldchange value of phosphorylation or protein expression between THY-10A62 treatment group and Vehicle group.

Our preceding study has confirmed that the increased phosphorylation of the FAK protein is a crucial factor in the development of liver cancer (14). To investigate the relationship between THY-10A62 treatment and phosphorylation levels of FAK, tumor tissues were harvested from PDX mice treated with THY-10A62 (15mg/kg), as well as from untreated PDX mice (Vehicle group). Protein phosphorylation microarray technology was employed to compare the differential protein phosphorylation profiles between these two groups (**Supplementary Figure 3A**). The analysis demonstrated a marked reduction in the expression of phosphorylated FAK protein in tumor tissues treated with THY-10A62 (15mg/kg) compared to the Vehicle group (**Figure 5**). These findings are consistent with our design rationale of THY-10A62 targeting the FAK protein structure.

In addition, the protein phosphorylation microarray identified 33 proteins that showed consistent trends in the phosphorylation and protein expression totally, including 21 up-regulated proteins and 12 down-regulated proteins. Among them, the phosphorylation levels of FAK (PTK2) downstream molecules, such as BRAF, RASGRF1, AKT1, Actin were decreased, while the phosphorylation levels of BAD was increased (**Figure 5**, **Supplementary Table 9**). To further validate these findings at the transcriptional level, we performed qPCR analysis on tumor tissues from PDX mice treated with THY-10A62 (15 mg/kg). The results showed significant downregulation of PTK2 (0.308-fold, p < 0.0001), BRAF (0.498-fold, p = 0.025), RASGRF1 (0.679-fold, p = 0.017), and the results showed slight downregulation of Actin (0.880-fold, p = 0.026) and AKT1 (0.870-fold, p = 0.048), while the RNA expression of BAD remained unchanged (1.040-fold, p > 0.05). These data support the transcriptional correlation with the observed protein phosphorylation changes (**Supplementary Figure 3E**, **Supplementary Table 10**).

**Figure 6:**
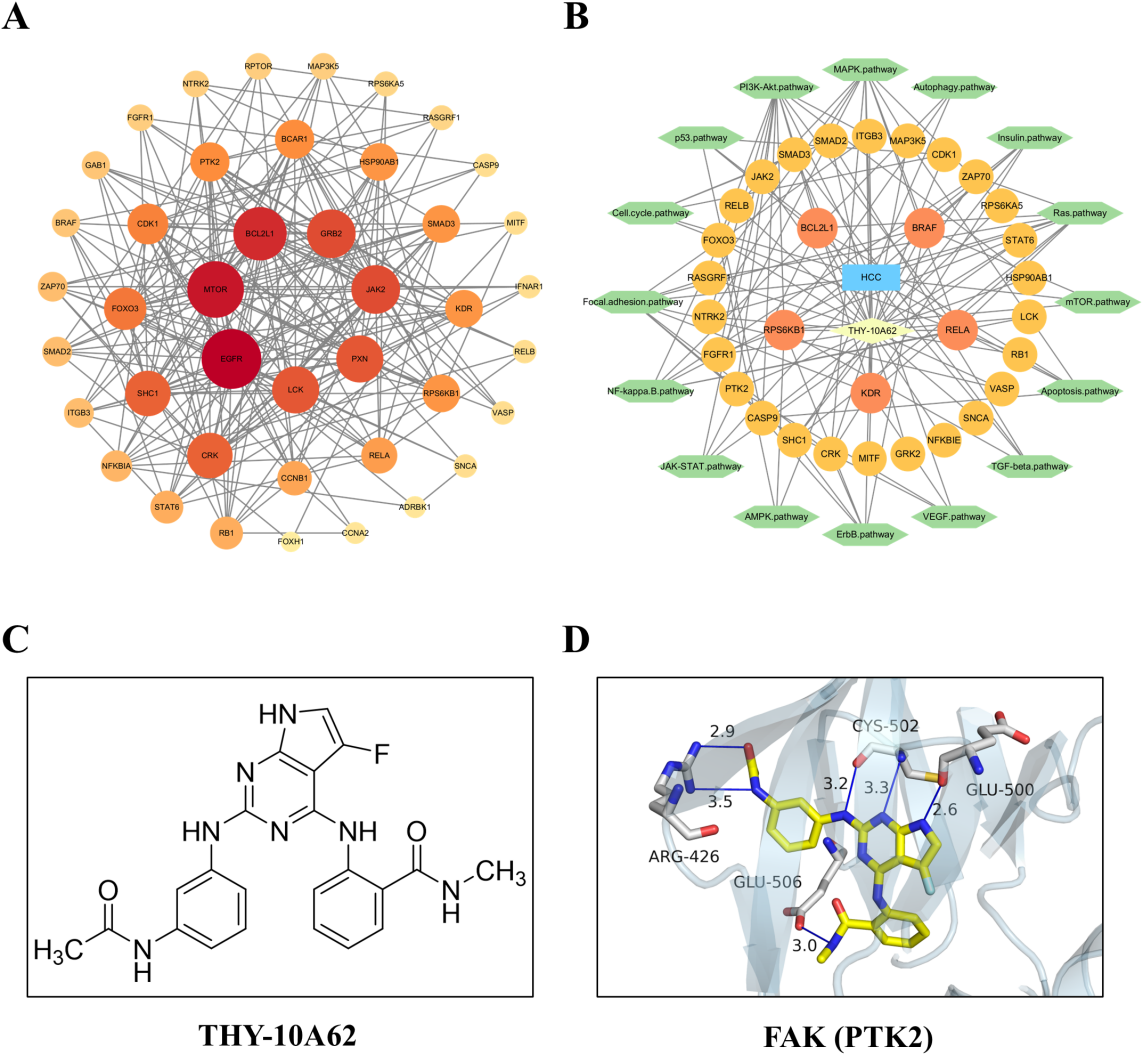
The function and interaction analysis of key differential expression genes. **(A)** the PPI network of shared genes related to HCC; **(B)** the PPI network of THY-10A62-HCC-target gene-pathway; **(C)** the molecular structure formular of THY-10A62; **(D)** The molecular docking simulation of THY-10A62 with FAK.

To investigate the molecular mechanism underlying THY-10A62’s inhibition effects on liver cancer proliferation, we employed network pharmacology methods combined with protein chip analysis. Subsequently, 33 differentially expressed proteins were integrated with 9392 hepatocellular carcinoma-related protein coding genes from the GeneCards database using a relevance score threshold (score >1). This resulted in a total of 32 common genes identified (**Supplementary Figure 4**). The STITCH online tool was employed to conduct a protein interaction network analysis for these 32 genes. Cytoscape 3.10.1 software was then utilized to visualize the PPI network mapping for target gene interactions (**Figure 6A**) and the relationships between THY-10A62/HCC-targeted genes/signaling pathway (**Figure 6B**). Based on the molecular structure formular of THY-10A62 (**Figure 6C**), the 3D structure was generated by PyMOL software. The molecular docking result demonstrated clearly interaction between THY-10A62 and FAK (PTK2) (PDB ID: 3BZ3; Energy: -10.2kcal) (**Figure 6D**).

### 3.6 FAK is significantly highly expressed in liver cancer tissues and is positively correlated with tumor prognosis

**Figure 7:**
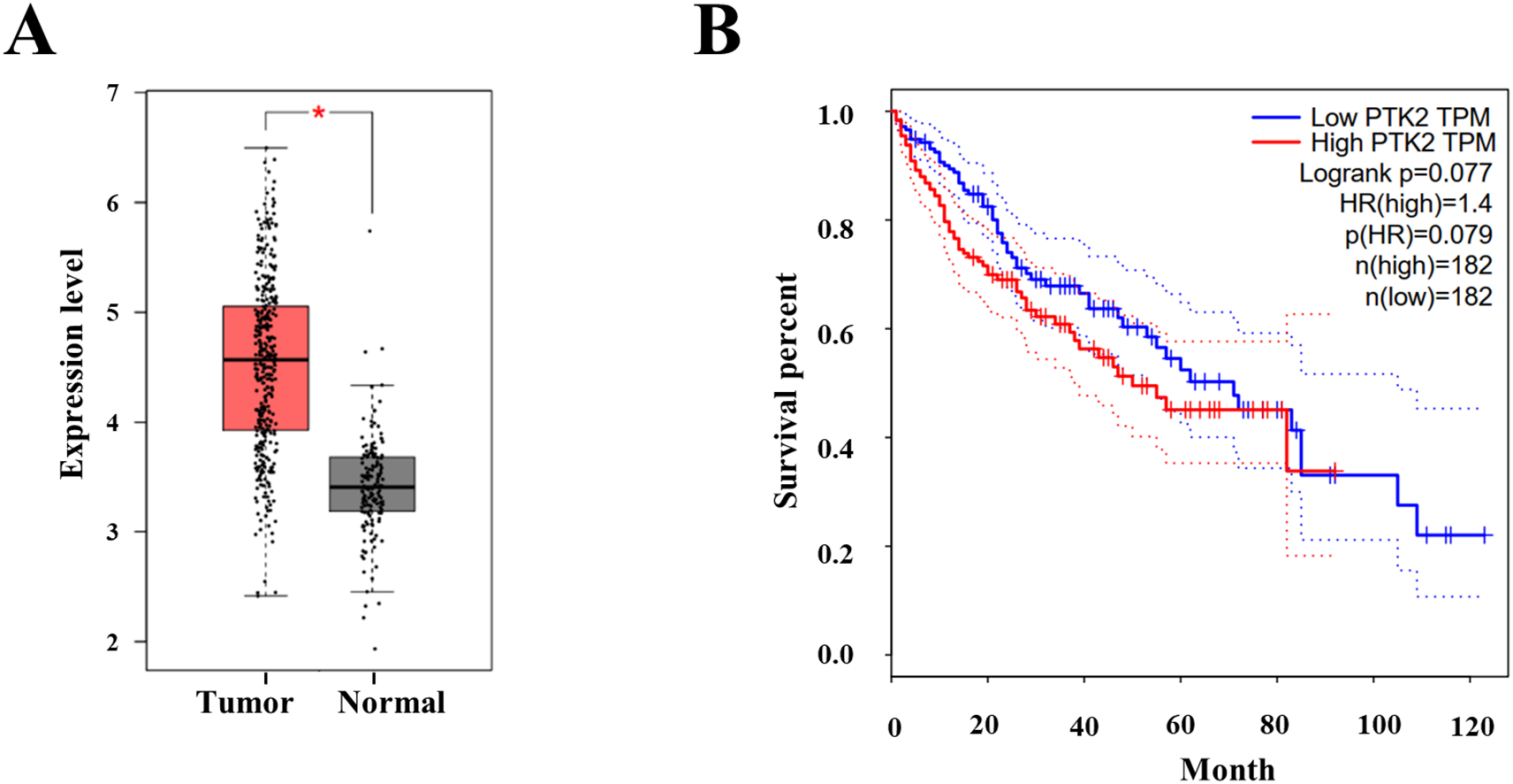
The protein expression and survival analysis of FAK (PTK2) in hepatocellular carcinoma. **(A)** The protein expression of FAK between Tumor (n=369) and adherence normal tissue (n=160); **(B)** The OS curve of high PTK2 (FAK) group and low PTK2 (FAK) group (data cited from GEPIA database). * represents p < 0.05.

We conducted RT-PCR to assess the expression levels of FAK gene in 95 pairs of HCC and adjacent non-cancerous tissue samples. The RNA expression of FAK (PTK2) was significantly overexpressed (foldchange > 2) in 71.5% (68/95) of the cancer tissues (**Supplementary Figure 5**). The down-regulated RNA expression (foldchange < 0.5) was detected in one case of liver cancer tissue when compared to the adjacent non-cancerous tissue. Then, we employed the GEPIA database to further investigate the RNA expression of FAK (PTK2) in liver cancer tissues. The results confirmed a marked overexpression of FAK in liver cancer (**Figure 7A**). Moreover, the results demonstrated that patients with high FAK expression level exhibited a significantly shorter overall survival (OS) compared to those with lower FAK expression (**Figure 7B**). These collective findings strongly suggest that FAK inhibitors, such as THY-10A62, as innovative therapeutic agents for the treatment of liver cancer.

## 4 Discussion

FAK is a 125 kDa cytoplasmic tyrosine kinase It encoded by the protein tyrosine kinase 2 (PTK2) gene (29, 30). FAK occupies adhesive sites between cells and the extracellular matrix, playing an important role in anchorage-dependent cell survival and integrin-mediated cell migration (31). It plays a key role in cellular signaling by integrating diverse extracellular inputs from transmembrane receptors, including growth factors, G protein-coupled receptors, and integrins cytokines, thereby initiating a various of intracellular signaling pathways involved in a broad spectrum of cellular activities (19, 32).

FAK plays a crucial role in regulating focal adhesion dynamics and turnover during the process of cell migration (14). FAK is overexpressed or hyperactivated in various tumor types (17, 33) and has identified as an independent risk factor for HCC (15). Our analysis using the GEPIA database further corroborated these findings. The protein expression level of FAK was up-regulated in 40% (13/31) tumor tissues (**Supplementary Figure 6**), including Cholangio carcinoma, Lymphoid Neoplasm Diffuse Large B-cell Lymphoma, Head and Neck squamous cell carcinoma, Stomach adenocarcinoma (STAD), Pancreatic adenocarcinoma (PAAD), Thymoma (THYM), Esophageal carcinoma, and Liver cancer (LIHC) (**Figure 7A**, **Supplementary Figures 6**, **7**). The overall survival (OS) analysis demonstrated that patients with high expression level of FAK exhibited a significantly shorter OS compared to those with lower expression level of FAK in LIHC, DLBC, PAAD, STAD and THYM tumors (**Figure 7B**, **Supplementary Figure 7**). This suggests that FAK inhibitors could represent a novel class of anticancer drugs effective against a range of cancer types.

Phosphorylated FAK along with other focal adhesion complex-related proteins, such as p130Cas and paxillin, is essential for the formation of focal adhesion and cell migration (32). In many tumor cells, the activation of FAK forms complexes with Src and p130Cas, promoting cell motility, invasion, cell cycle progression, survival, angiogenesis, and EMT through downstream signaling pathways. These processes contribute to tumor growth and metastasis (18). In recent years, the recognition of FAK as a novel target for anti-cancer drug development has opened new avenues for inhibiting tumor progression and metastasis (34).

FAK consists of three domains: the N-terminal FERM (Focal Adhesion Targeting) domain, the central kinase domain, and the C-terminal domain. The N-terminal FERM domain comprises three subdomains, forming a cloverleaf-like structure. This structure mediates protein-protein interactions as well as protein-lipid interactions. The central kinase domain shares significant structural and functional similarity to other tyrosine kinases. The activation loop of the kinase domain contains tyrosine residues Y576 and Y577, which are essential for regulating its catalytic activity. The C-terminal domain consists of the FAT and PRRs structural domains, which participate in numerous protein-protein interactions. FAK is usually located in the cytoplasm in an inactive state, with the FERM domain directly interacting with the kinase domain, inhibiting its catalytic activity. Upon binding to integrin family members (ECM), specific growth factors or G-proteins, FAK undergoes autophosphorylation and becomes fully activated. Furthermore, alterations in FAK conformation and changes in intracellular physicochemical properties can also activate FAK (35).

According to incomplete statistics, there are currently over twenty FAK inhibitors in various stages of development, with most of them in pre-clinical research. Among them, GSK and Pfizer have the most significant presence in this field, with three compound s each. Others include Amplia Therapeutics, Boehringer Ingelheim, Novartis, and more. In addition to these, domestic companies in China, such as Sailun Tai, Haichuang, Yasheng, and Yingshi Biotech, are also actively engaged in this field. The focus of indication development mainly centers on oncology (36, 37). However, the use of novel FAK inhibitors in liver cancer therapy has yet to be reported, and our team has been committed to the research work in this area.

In 2022, a study published in Science Translational Medicine reported the successful prevention of metastasis formation in mice by implanting brain metastatic breast cancer cells into mice and treating them with FAK inhibitors developed by Pfizer and Verastem (38). Furthermore, by using imaging techniques to track tumor progression, it was shown that FAK inhibitors could suppress the further growth of tumors. This suggests that FAK may play a crucial role in inhibiting breast cancer brain metastasis and holds promise for improving prognosis of patients with metastatic breast cancer. Our study also demonstrates that THY-10A62 has the capability to inhibit the infiltration of liver cells (**Figure 2**). In 2021, the Edinburgh Cancer Research Center contributed to the field by publishing an article in Nat Rev Cancer (31). Through an analysis of preclinical data, they concluded that FAK inhibitors represent a potential strategy to overcome resistance to chemotherapy, radiation therapy, or targeted treatment.

In this study, the results from phosphorylated protein chip analysis demonstrated that THY-10A62 significantly attenuated the phosphorylation and expression levels of FAK (PKT2) (**Figure 5**, **Supplementary Table 9**). Furthermore, we observed a significant reduction in the phosphorylation and expression levels of PDGFRB, RASGRF1, and BRAF proteins within the Focal Adhesion signaling pathway (**Figure 5**), which has been reported that elevated expression of these proteins promotes tumor progression and is strongly associated with poor prognosis in cancer patients (39, 40). Artificially inhibiting their expression can effectively impair chemotactic and invasive functions of tumor cells, thereby achieve therapeutic effects or improve patient prognosis (39–41). Additionally, we noted a substantial increase in the phosphorylation level of BAD protein. As a pro-apoptotic molecule, increased phosphorylation of BAD can enhance tumor cell apoptosis (42). Our findings suggest that THY-10A62 (15mg/kg) suppresses FAK (PTK2) protein phosphorylation and expression in mice while potentially upregulating BAD protein phosphorylation through FAK/BRAF pathway to promote tumor cell apoptosis, through FAK/AKT pathway to inhibit tumor motility or through FAK/RASGRF1/Actin pathway to inhibit tumor cell proliferation, thus exerting the anti-liver cancer effect. In future studies, we aim to provide further evidence supporting this novel perspective and offer new directions for developing innovative technologies for liver cancer treatment.

Our previous work based on functional genomics technologies revealed that SCARA5 suppresses the FAK tyrosine phosphorylation cascade along with its associated downstream signaling cascades. Decreased levels of SCARA5 in HCC may potentially induce the activation of the signaling cascade linked to focal adhesion complexes. This implied that SCARA5 suppression in HCC cells, potentially induced by allelic loss, DNA hypermethylation in the promoter CpG island, histone modifications, could promote cell proliferation and other malignant phenotypes in HCC. Consequently, the inhibition of FAK protein expression and tyrosine phosphorylation can suppress the capacity for proliferation of liver cancer cells, as well as their ability to metastatic capacity *in vivo* (14).

THY-10A62 was a novel FAK inhibitor with a 5-fluoro-7H-pyrrolo[2,3-d]pyrimidine-2,4-diamine core (**Supplementary Figure 1**). It was designed and optimized according to the E-pharmacophores generated by docking a library of 667 fragments into the ATP pocket of the co-crystal complex of FAK (PDB ID: 3BZ3) and PF-562271 (31, 43). As shown in **Figure 3** of our previous study (27), molecular docking analysis revealed that THY-10A62 formed three hydrogen bonds with the hinge motif of FAK: two with Cys502 (via the nitrogen at the 1-position and the amino group at the 2-position of the pyrimidine ring) and one with Glu500 (via the NH group of the fused pyrrole ring). These interactions mimic the binding mode of PF-562271 and anchor the molecule firmly within the kinase domain. In addition, the pyrrole ring engages in hydrophobic interactions with the gatekeeper residue Met499, further stabilizing the binding. Notably, the phenylamino group at the 4-position adopts a distinct orientation compared to the pyridine ring of PF-562271, with the NH group of its methyl carbamoyl substituent forming an additional hydrogen bond with Glu506, enhancing binding affinity (-10.2 *vs*. -8.9 kcal/mol for PF-562271) (27). These structural optimizations contribute to THY-10A62’s superior activity. Moreover, tissue distribution analysis (**Supplementary Figure 2**) shows higher tumor accumulation than PF-562271, likely due to improved membrane permeability from its lipophilic fluorinated structure. These advantages enable competitive and reversible inhibition of FAK phosphorylation, with sustained plasma levels supporting its antitumor efficacy *in vivo*.

Notably, although THY-10A62 levels were higher in lung homogenates (**Supplementary Figure 2**), the H-score in lung tissues showed only a modest reduction (**Figure 4**). This apparent discrepancy may be explained by several factors. First, LC-MS quantifies total drug levels in tissue, but only the unbound, bioactive fraction that penetrates tumor foci contributes to pharmacodynamic effects (44). Second, the lung exhibits high metabolic and efflux activity, particularly via cytochrome P450 enzymes and ABC transporters, which may reduce the intratumoral concentration of the active compound (45). Third, baseline expression of key biomarkers such as p-FAK and EpCAM is typically lower and more heterogeneous in lung micrometastases compared to primary liver tumors (46, 47). Lastly, lung tissue sections often contain scattered micrometastases within normal parenchyma, diluting the immunohistochemical signal, whereas liver sections are largely tumor-rich, making changes in H-score more readily detectable.

Our previous study revealed that THY-10A62 exhibited superior anti-proliferative effects on two cell lines (SMMC7721 and YY8103) compared to PF-562271. In an *in vivo* evaluation using the SMMC7721 xenograft tumor model, a dose of 30 mg/kg of THY-10A62 significantly suppressed tumor growth, with TGI% of 78.6%, whereas PF-562271 achieved a TGI% of 54.1% (27). In this study, utilizing the HCC-LM3 CDX model, THY-10A62 demonstrated the higher TGI% (45.6%) compared to Sorafenib and PF-562271 (**Figure 2**, **Table 1**). Furthermore, in PDX model, THY-10A62 also displayed greater tumor inhibitory effects than PF-562271 at the same dose of 15 mg/kg (**Figure 3C**, **Supplementary Table 8**). Therefore, these findings suggest that THY-10A62 demonstrates enhanced liver cancer inhibition compared to PF-562271, potentially due to its favorable membrane permeability and potential for improved interaction with FAK. Additionally, the network pharmacology and protein phosphorylation chip technology indicated that THY-10A62 effectively reduced the phosphorylation level of FAK protein and concurrently modulated the phosphorylation levels of downstream effectors RASGRF1 and BRAF in the FAK signaling pathway (**Figure 5**, **Supplementary Figure 3**, **Supplementary Table 9**), representing a potential regulatory mechanism by which THY-10A62 suppresses liver cancer cell proliferation.

In summary, our study demonstrates that THY-10A62 exhibits potent antitumor activity against HCC *in vivo*, showing superior tumor growth inhibition compared to the vehicle, PF-562271, and sorafenib treatment groups. Phospho-protein antibody array analysis further revealed that THY-10A62 reduces the phosphorylation of key proteins within the FAK/RASGRF1 and/or FAK/BRAF signaling pathways, potentially contributing to its antiproliferative effects.

Future investigations will focus on systematically evaluating the clinical potential of THY-10A62, including identification of specific phosphorylation sites on FAK inhibited by the compound, characterization of downstream signaling changes, and comprehensive pharmacokinetics-pharmacodynamics analyses. In addition, the efficacy of THY-10A62 will be further validated in lung metastasis and other tumor models to gain a deeper understanding of its pharmacological mechanisms and therapeutic relevance.

## Data Availability

The datasets presented in this study can be found in online repositories. The names of the repository/repositories and accession number(s) can be found in the article/**Supplementary Material**.
