# Supplementary material for: Evaluation of anti-liver cancer activity and anticancer mechanism of one novel small molecule compound (THY-10A62) targeting FAK pathway
Source: Front Oncol. 2025 Sep 1;15:1498005. doi: 10.3389/fonc.2025.1498005 (PMC12433887; doi:10.3389/fonc.2025.1498005)
Supplement: Supplementary file 1 [file DataSheet1.docx]

***Supplementary Material***

Evaluation of anti-liver cancer activity and anticancer mechanism of one novel small molecule compound (THY-10A62) targeting FAK pathway

**Wanqiu Huang^1^, Rong Zou^1^, Jie Xu^1^, Yuliang Deng^1^, Dongping Zhang^2^, Yiguo Hu^3*^, Qian Zhang^2*^, Jian Huang^1*^, Zhaoqi Zhang^1, 4*^**

^1^Key Laboratory of Systems Biomedicine (Ministry of Education), Shanghai Centre for Systems Biomedicine, Shanghai Jiao Tong University, Shanghai, 200240, China

^2^Department of Medicinal Chemistry, School of Pharmacy, Fudan University, Shanghai, 201203, China

^3^Department of Thyroid Surgery and National Clinical Research Center for Geriatrics, State Key Laboratory of Biotherapy and Cancer Center, West China Hospital, Sichuan University, and Collaborative Innovation Center for Biotherapy, Chengdu, 610041, China

^4^Department of Breast and Thyroid Surgery, Shanghai General Hospital, Shanghai Jiao Tong University School of Medicine, Shanghai, 200080, China

*** Correspondence:**Zhaoqi Zhang (drzzq@foxmail.com)
Jian Huang (jianhuang@sjtu.edu.cn)
Qian Zhang (zhangqian511@shmu.edu.cn)
Yiguo Hu (huyuguo99@126.com)

Keywords: Liver cancer, small molecule compound, THY-10A62, FAK inhibitor, mechanism, signal pathway

# Supplementary Figures


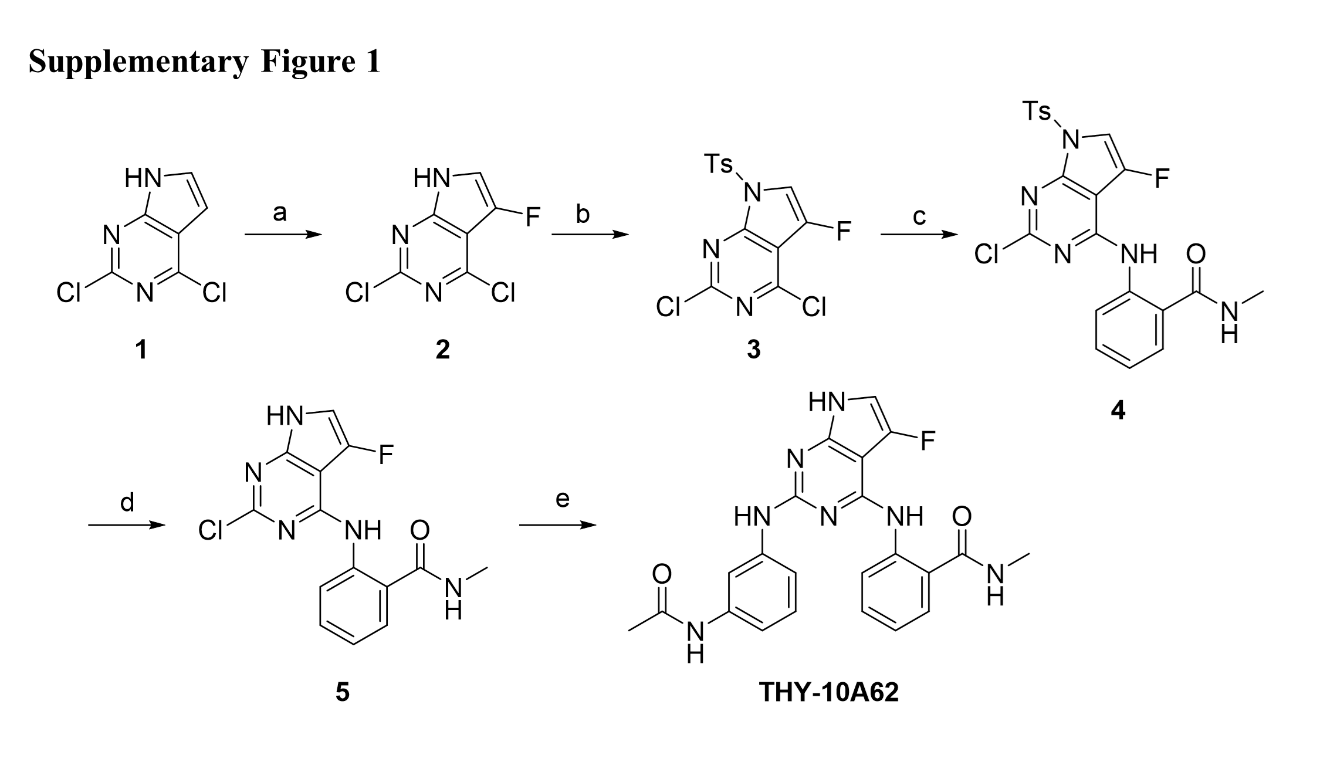


**Supplementary Figure 1.** Synthetic route for THY-10A62.

Reagents and conditions: (a) Selectflour, AcOH, MeCN, 70 ° C, overnight, 50%; (b) TsCl, TEA, DMAP, DCM, rt, 2 h, 72%; (c) 2-amino-*N*-methylbenzamide, DIPEA, MeCN, reflux, 40 h, 49%; (d) TBAF, THF, reflux, 12 h, 70%; (e) *N*-(3-aminophenyl)acetamide, Pd_2_(dba)_3_, Xphos, K_2_CO_3_, *t*-BuOH, 100 °C, 15 h, 37%.

The synthetic route: 2,4-Dichloro-7H-pyrrolo[2,3-d] pyrimidine (1) was employed as the starting material and reacted with Selectfluor to introduce a fluorine atom into the 5-position (2). The nitrogen atom in the pyrrole ring of 2 was sulfonylated by stirring the substrate with p-toluenesulfonyl chloride in dichloromethane at room temperature to provide 3, which was then reacted with 2-amino-N-methylbenzamide, undergoing nucleophilic substitution to introduce the side chain at the 4-position (4). After removal of the protecting group by TBAF, the desired product was obtained via the Buchwald-Hartwig reaction of 5 with N-(3-aminophenyl) acetamide. The structure was identified by 1H-NMR, 13C-NMR and HR-MS spectra.

Please see the detail synthetic route: Scheme 5 in Eur J Med Chem. 2021:223:113670.


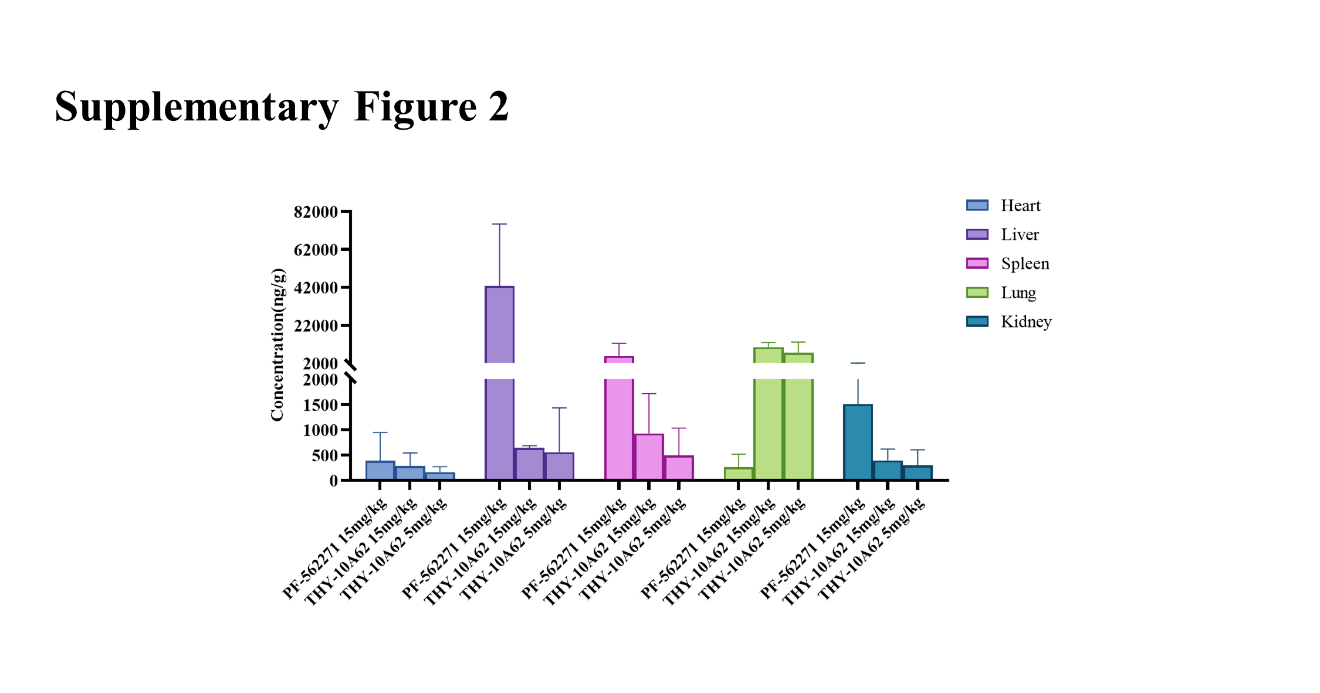


**Supplementary Figure 2.** The drug distribution in main organs of PDX mice after treated with THY-10A62. The organs include heart, liver, spleen, lung and kidney.


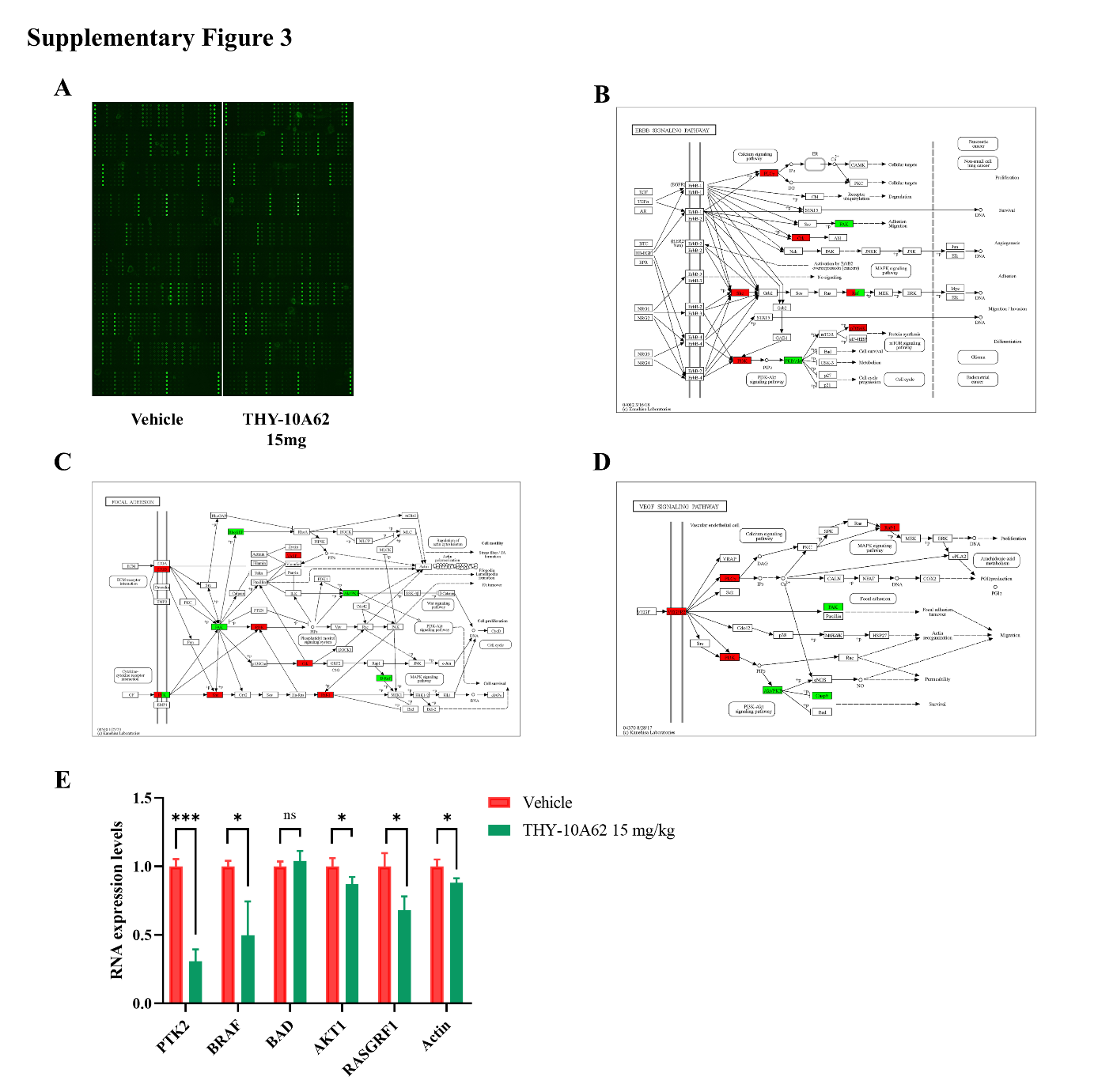


**Supplementary Figure 3.** The protein phosphorylation microarray results of PDX tumor mass between THY-10A62 15mg/kg group and Vehicle group. (A) protein phosphorylation microarray detection result; the difference of partial protein phosphorylation in ERBB signal pathway (B), Focal adhesion signal pathway (C) and VEGF signal pathway (D). (E) qPCR validation of mRNA expression changes in FAK (PTK2) and downstream genes (BRAF, RASGRF1, AKT1, BAD, and Actin) in tumor tissues of PDX mice treated with THY-10A62 (15 mg/kg) versus vehicle control. Red highlight indicates up-regulated phosphorylation, and green highlight indicates down-regulated phosphorylation. Experiments were conducted in three independent replicates. Data are presented as mean ± SEM. *p < 0.05; **p < 0.01; ***p < 0.001 vs. Vehicle group. ns indicates no significant difference.


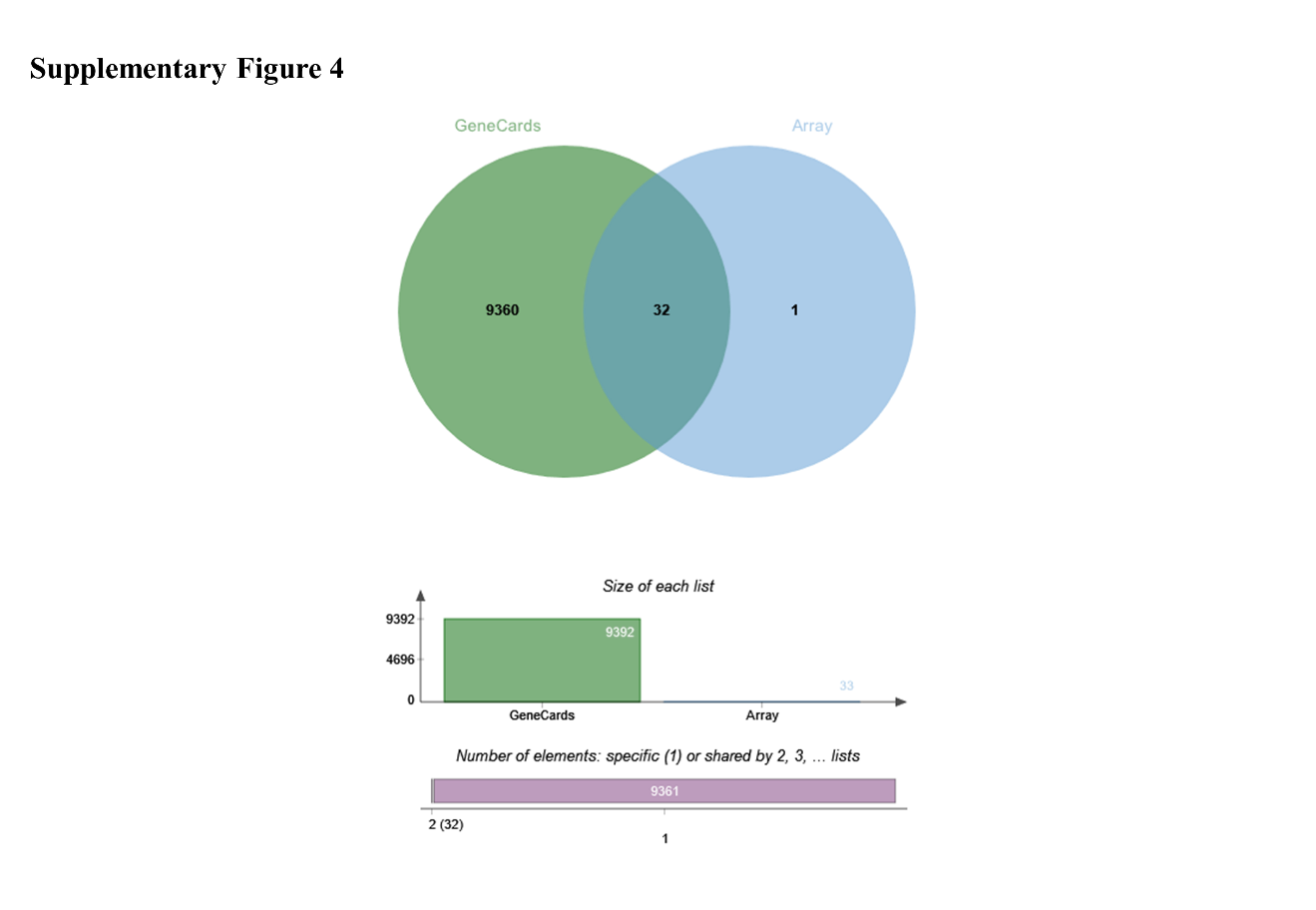


**Supplementary Figure 4.** The shared genes analysis of DEGs from protein phosphorylation microarray and hepatocellular carcinoma-related genes from GeneCards database.


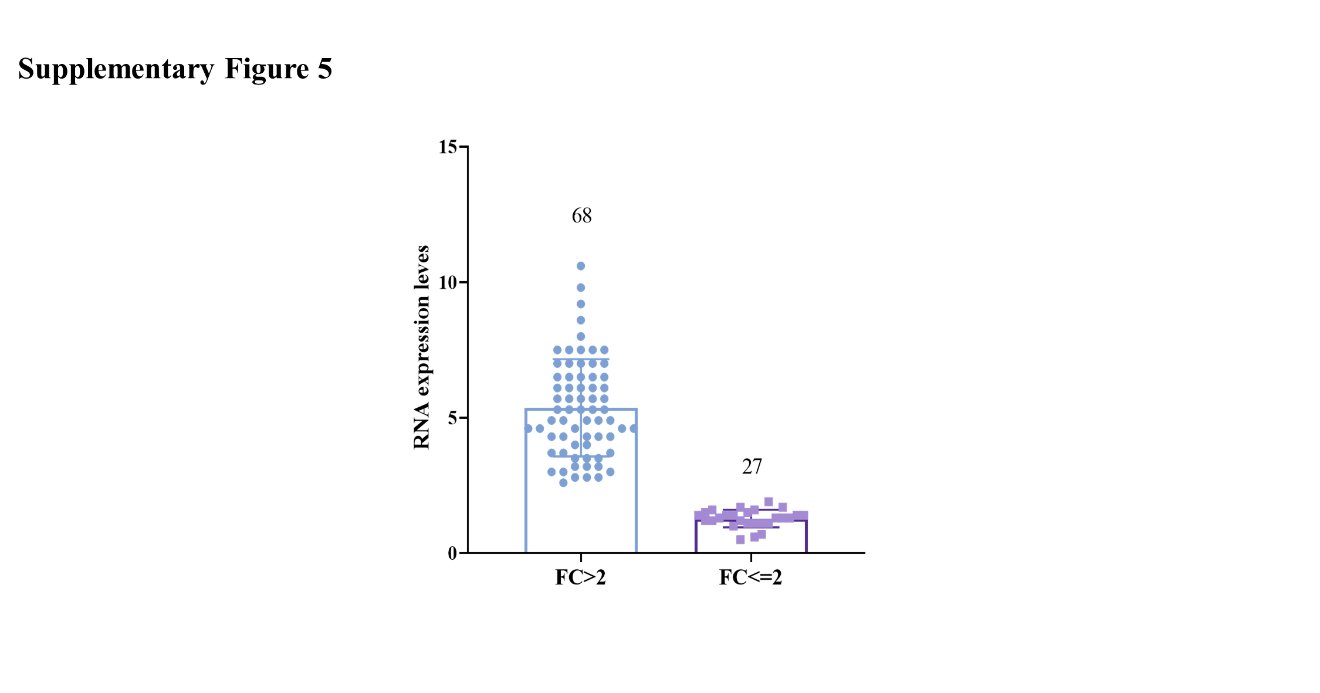


**Supplementary Figure 5.** The expression levels of FAK in 95 pairs of liver cancer and adjacent non-cancerous tissue samples. Foldchange value >2 as the grouping threshold.


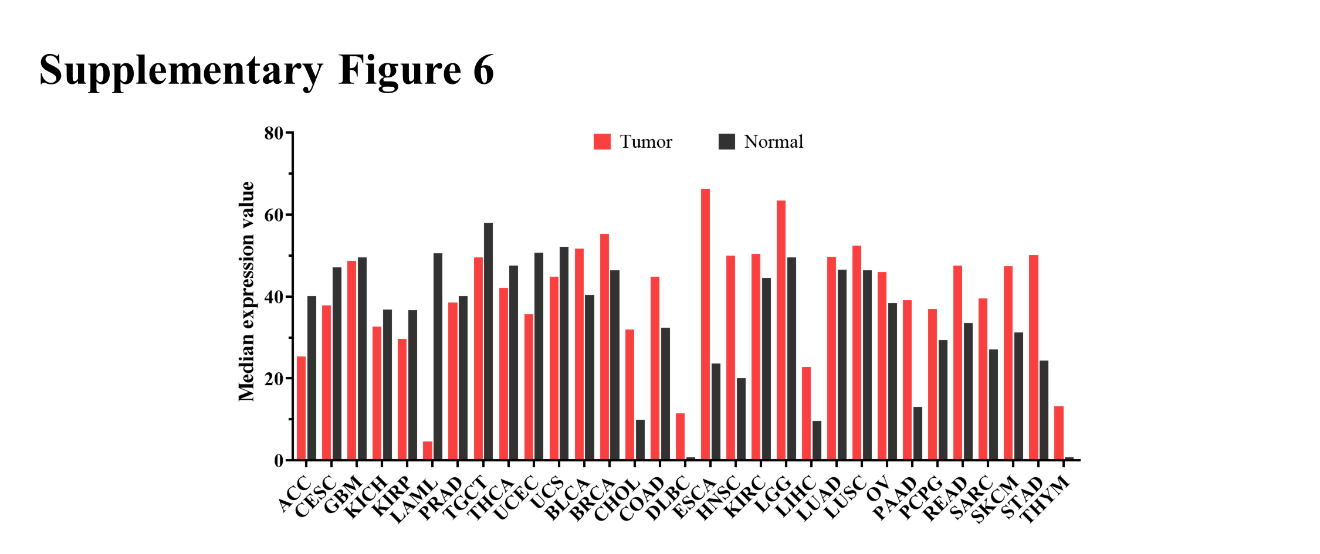


**Supplementary Figure 6.** The statistics of FAK (PTK2) protein expression in 31 human tumors data cited from GEPIA database).


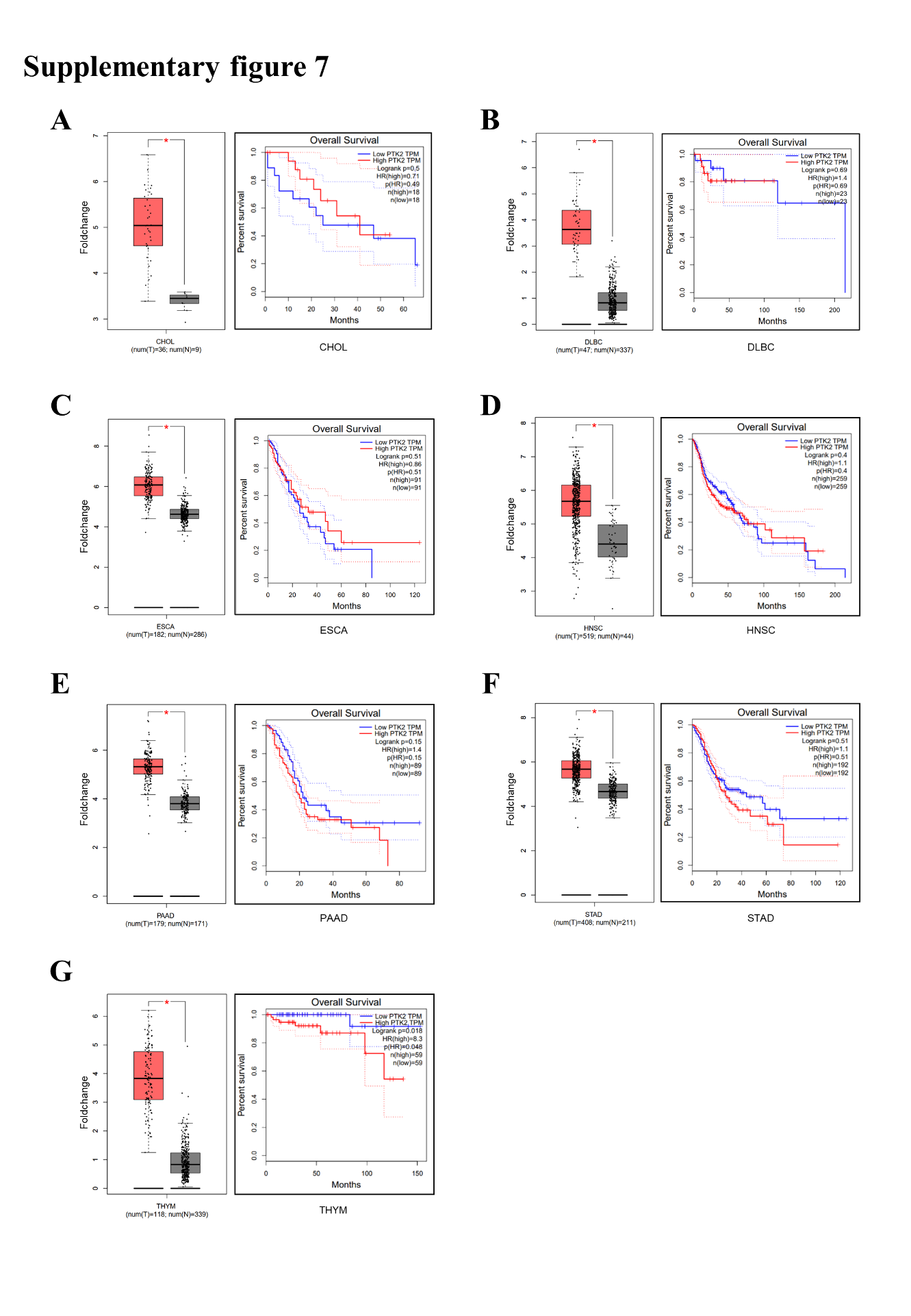


**Supplementary Figure 7.** The protein expression and overall survival analysis of FAK in (A) CHOL, (B) DLBC, (C) ESCA, (D) HNSC, (E) PAAD, (F) STAD and (G) THYM. Note: CHOL-Cholangio carcinoma, DLBC-Lymphoid Neoplasm Diffuse Large B-cell Lymphoma, ESCA-Esophageal carcinoma, HNSC-Head and Neck squamous cell carcinoma, PAAD-Pancreatic adenocarcinoma, STAD-Stomach adenocarcinoma, THYM-Thymoma (data cited from GEPIA database).


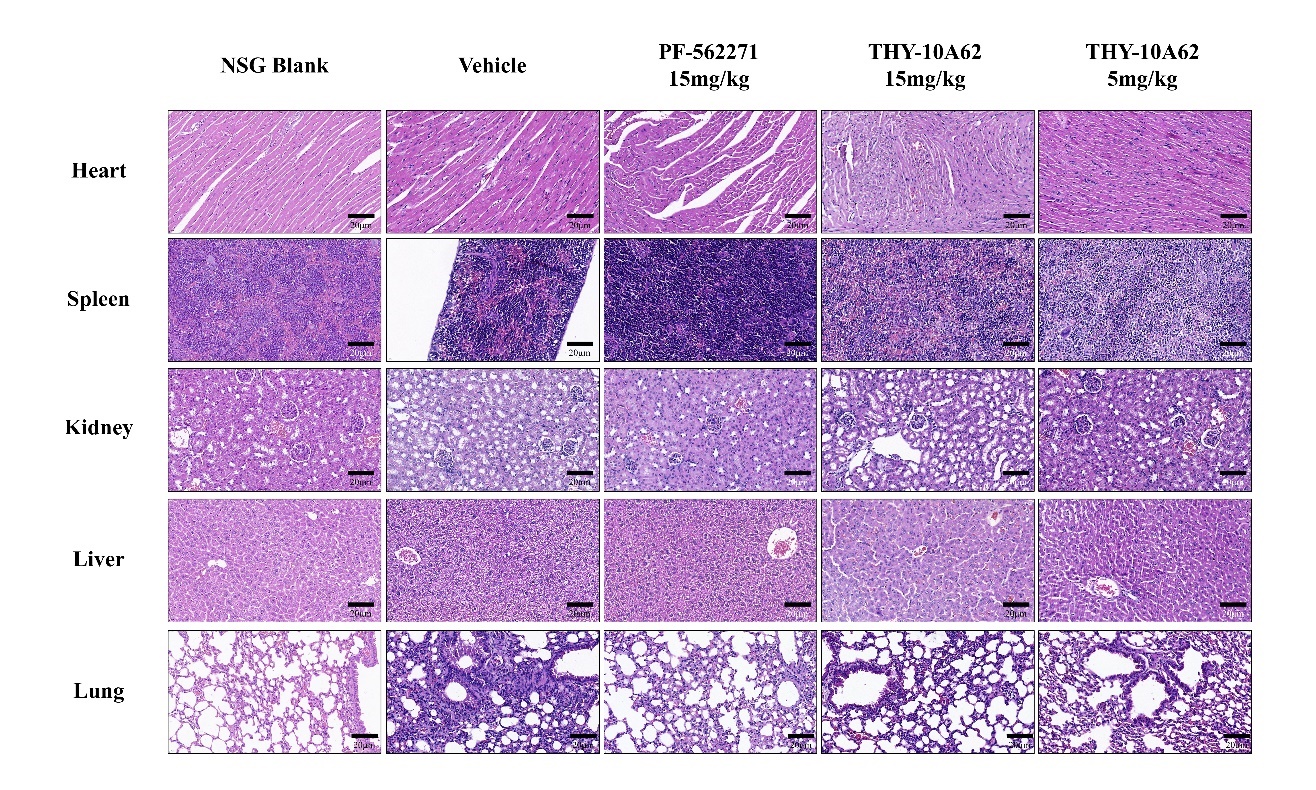


**Supplementary Figure 8.** The HE staining for the tissue organs of heart, spleen, kidney, liver and, lung following treatment with PF-562271 and THY-10A62.

# Supplementary Tables

**Supplementary Table 1. Body weight changes of female ICR mice on the 3^rd^ day**

| **Group** | **Administration dose of THY-10A62 (mg/kg)** | **Average weight on D0 (g)** | **Average weight on 3^rd^ day (g)** | **Number of mice initial** | **Number of mice surviving on 3^rd^ day** | **Weigh change rate (%)** |
| --- | --- | --- | --- | --- | --- | --- |
| Group1 | 100 | 24.90 | NA | 3 | 0 | NA |
| Group2 | 90 | 23.70 | NA | 3 | 0 | NA |
| Group3 | 80 | 25.73 | NA | 3 | 0 | NA |
| Group4 | 70 | 25.94 | NA | 5 | 0 | NA |
| Group5 | 60 | 25.72 | 27.20 | 5 | 1 | 4.21% |
| Group6 | 54 | 25.18 | 25.27 | 10 | 3 | 0.36% |
| Group7 | 50 | 27.04 | 26.97 | 10 | 4 | -0.26% |
| Group8 | 45 | 25.71 | 25.93 | 10 | 7 | 0.86% |
| Group9 | 0 | 23.97 | 24.67 | 3 | 3 | 2.92% |

**Note:** The day of group administration is D0. NA indicates that the data was not available.

| **Group** | **Administration dose of THY-10A62 (mg/kg)** | **Average weight on D0 (g)** | **Average weight on 3^rd^ day (g)** | **Number of mice initial** | **Number of mice surviving on 3^rd^ day** | **Weigh change rate (%)** |
| --- | --- | --- | --- | --- | --- | --- |
| Group1 | 90 | NA | NA | 3 | 0 | NA |
| Group2 | 80 | NA | NA | 3 | 0 | NA |
| Group3 | 70 | 34.64 | 33.8 | 5 | 1 | -2.42% |
| Group4 | 63 | 38.54 | 36.9 | 5 | 1 | -4.26% |
| Group5 | 56 | 33.40 | 33.60 | 5 | 3 | 0.60% |
| Group6 | 45 | 34.04 | 34.00 | 5 | 4 | -0.12% |
| Group7 | 36 | 33.07 | 34.43 | 3 | 3 | 4.11% |
| Group8 | 30 | 32.80 | 34.17 | 3 | 3 | 4.18% |
| Group9 | 0 | 34.30 | 35.13 | 3 | 3 | 2.42% |

**Supplementary Table 2. Body weight changes of male ICR mice on the 3^rd^ day**

**Note:** The day of group administration is D0. NA indicates that the data was not available.

**Supplementary Table 3. Statistics of LD_50_ of THY-10A62 in female mice**

| **Cell counting and residuals** | | | | | | |
| --- | --- | --- | --- | --- | --- | --- |
| **No.** | **Dosage** | **No. of Subjects** | **Measured Response** | **Expected Response** | **Residual** | **Probability**  **(PROBIT)** |
| 1 | 100.000 | 3 | 3 | 3.000 | 0.000 | 1.000 |
| 2 | 90.000 | 3 | 3 | 3.000 | 0.000 | 1.000 |
| 3 | 80.000 | 3 | 3 | 2.998 | 0.002 | 0.999 |
| 4 | 70.000 | 5 | 5 | 4.929 | 0.071 | 0.986 |
| 5 | 60.000 | 5 | 4 | 4.367 | -0.367 | 0.873 |
| 6 | 54.000 | 10 | 7 | 6.963 | 0.037 | 0.696 |
| 7 | 50.000 | 10 | 6 | 5.378 | 0.622 | 0.538 |
| 8 | 45.000 | 10 | 3 | 3.340 | -0.340 | 0.334 |

| **Confidence Limit (CI)** | | | |
| --- | --- | --- | --- |
| **Probability**  **(PROBIT)** | **95% CI of Dosage** | | |
|  | **Estimate** | **Lower limit** | **Upper limit** |
| .010 | 26.890 | -49.834 | 37.818 |
| .020 | 29.492 | -39.110 | 39.352 |
| .030 | 31.143 | -32.313 | 40.333 |
| .040 | 32.385 | -27.204 | 41.075 |
| .050 | 33.395 | -23.053 | 41.683 |
| .060 | 34.255 | -19.522 | 42.203 |
| .070 | 35.008 | -16.429 | 42.662 |
| .080 | 35.683 | -13.662 | 43.075 |
| .090 | 36.297 | -11.147 | 43.453 |
| .100 | 36.862 | -8.835 | 43.803 |
| .150 | 39.202 | 0.712 | 45.279 |
| .200 | 41.061 | 8.254 | 46.496 |
| .250 | 42.657 | 14.675 | 47.592 |
| .300 | 44.089 | 20.378 | 48.638 |
| .350 | 45.417 | 25.579 | 49.692 |
| .400 | 46.677 | 30.392 | 50.813 |
| .450 | 47.895 | 34.865 | 52.082 |
| **.500** | **49.095** | **38.969** | **53.628** |
| .550 | 50.294 | 42.604 | 55.645 |
| .600 | 51.513 | 45.628 | 58.363 |
| .650 | 52.773 | 48.000 | 61.925 |
| .700 | 54.100 | 49.856 | 66.324 |
| .750 | 55.533 | 51.401 | 71.528 |
| .800 | 57.128 | 52.813 | 77.631 |
| .850 | 58.987 | 54.242 | 84.963 |
| .900 | 61.327 | 55.867 | 94.360 |
| .910 | 61.892 | 56.242 | 96.648 |
| .920 | 62.506 | 56.644 | 99.139 |
| .930 | 63.181 | 57.080 | 101.883 |
| .940 | 63.935 | 57.560 | 104.954 |
| .950 | 64.795 | 58.102 | 108.463 |
| .960 | 65.805 | 58.730 | 112.594 |
| .970 | 67.047 | 59.494 | 117.681 |
| .980 | 68.697 | 60.497 | 124.456 |
| .990 | 71.299 | 62.057 | 135.154 |

**Supplementary Table 4. Statistics of LD_50_ of THY-10A62 in male mice**

| **Cell counting and residuals** | | | | | | |
| --- | --- | --- | --- | --- | --- | --- |
| **No.** | **Dosage** | **No. of Subjects** | **Measured Response** | **Expected Response** | **Residual** | **Probability** |
| 1 | 90.000 | 3 | 3 | 2.995 | 0.005 | 0.998 |
| 2 | 80.000 | 3 | 3 | 2.939 | 0.061 | 0.980 |
| 3 | 70.000 | 5 | 4 | 4.380 | -0.380 | 0.876 |
| 4 | 63.000 | 5 | 4 | 3.513 | 0.487 | 0.703 |
| 5 | 56.000 | 5 | 2 | 2.319 | -0.319 | 0.464 |
| 6 | 45.000 | 5 | 1 | 0.712 | 0.288 | 0.142 |
| 7 | 36.000 | 3 | 0 | 0.092 | -0.092 | 0.031 |
| 8 | 30.000 | 3 | 0 | 0.024 | -0.024 | 0.008 |

| **Confidence Limit (CI)** | | | |
| --- | --- | --- | --- |
| **Probability**  **(PROBIT)** | **Probability** | | |
|  | **Estimate** | **Lower limit** | **Upper limit** |
| .010 | 30.880 | -13.232 | 42.430 |
| .020 | 33.943 | -5.467 | 44.483 |
| .030 | 35.887 | -0.561 | 45.806 |
| .040 | 37.349 | 3.116 | 46.815 |
| .050 | 38.538 | 6.097 | 47.645 |
| .060 | 39.551 | 8.626 | 48.361 |
| .070 | 40.438 | 10.837 | 48.995 |
| .080 | 41.233 | 12.809 | 49.569 |
| .090 | 41.956 | 14.598 | 50.097 |
| .100 | 42.621 | 16.238 | 50.589 |
| .150 | 45.376 | 22.960 | 52.695 |
| .200 | 47.565 | 28.194 | 54.478 |
| .250 | 49.443 | 32.572 | 56.119 |
| .300 | 51.130 | 36.380 | 57.716 |
| .350 | 52.693 | 39.769 | 59.337 |
| .400 | 54.176 | 42.823 | 61.035 |
| .450 | 55.611 | 45.593 | 62.865 |
| **.500** | **57.023** | **48.110** | **64.873** |
| .550 | 58.435 | 50.402 | 67.107 |
| .600 | 59.870 | 52.496 | 69.611 |
| .650 | 61.353 | 54.431 | 72.429 |
| .700 | 62.916 | 56.253 | 75.617 |
| .750 | 64.602 | 58.016 | 79.259 |
| .800 | 66.481 | 59.794 | 83.501 |
| .850 | 68.670 | 61.688 | 88.623 |
| .900 | 71.424 | 63.886 | 95.253 |
| .910 | 72.090 | 64.395 | 96.876 |
| .920 | 72.813 | 64.940 | 98.648 |
| .930 | 73.607 | 65.530 | 100.604 |
| .940 | 74.495 | 66.181 | 102.799 |
| .950 | 75.507 | 66.912 | 105.312 |
| .960 | 76.696 | 67.759 | 108.276 |
| .970 | 78.159 | 68.784 | 111.937 |
| .980 | 80.102 | 70.126 | 116.824 |
| .990 | 83.166 | 72.201 | 124.567 |

**Supplementary Table 5. Statistics of tumor weight of CDX mice after drug treatment (g)**

| **Treatment** | **D1** | **D4** | **D7** | **D10** | **D13** | **D16** |
| --- | --- | --- | --- | --- | --- | --- |
| Vehicle | 117.6±14.2 | 186.2±19.4 | 287.7±49.3 | 472.9±126.3 | 670.3±138.4 | 867.9±120.9 |
| THY-10A62  5 mg/kg | 117.5±14.3 | 168.4±30.0 | 231.0±32.4^*^ | 345.5±61.8^*^ | 515.7±68.2^*^ | 689.4±74.7^*^ |
| THY-10A62  10 mg/kg | 117.9±13.9 | 163.5±27.1 | 211.6±37.4^**^ | 315.9±55.9^**^ | 447.9±88.6^**^ | 620.7±110.2^**^ |
| THY-10A62  15 mg/kg | 117.6±13.9 | 143.0±20.0^**^ | 172.5±29.9^***^ | 253.3±30.0^***^ | 353.2±71.5^***^ | 471.7±112.8^***^ |
| Sorafinib | 118.4±15.1 | 172.1±30.5 | 239.4±33.1^*^ | 362.9±64.8^*^ | 532.3±81.6^*^ | 719.3±70.5^*^ |
| PF-562271 | 118.6±14.7 | 170.0±29.8 | 233.1±31.6^*^ | 348.6±62.1^*^ | 497.5±76.3^*^ | 663.4±123.9^**^ |

**Note:** The day of group administration is D1. Data are presented as mean ± SEM. *p < 0.05; **p < 0.01; ***p < 0.001 vs. Vehicle group.

**Supplementary Table 6. Body weight of PDX mice after drug treatment (g)**

| **Treatment** | **D0** | **D3** | **D6** | **D9** | **D12** |
| --- | --- | --- | --- | --- | --- |
| Vehicle | 18.5±1.8 | 18.1±1.0 | 18.2±1.0 | 18.9±0.5 | 19.4±0.6 |
| THY-10A62  15 mg/kg | 19.1±1.5 | 18.3±1.8 | 18.7±1.7 | 19.0±0.7 | 19.9±0.5 |
| THY-10A62  5 mg/kg | 19.2±0.8 | 18.7±0.7 | 19.1±0.8 | 19.4±0.3 | 19.9±0.2 |
| PF-562271  15 mg/kg | 18.5±1.1 | 18.2±1.1 | 18.6±1.4 | 18.9±0.6 | 19.1±0.5 |

**Note:** The day of group administration is D0. Data are presented as mean ± SEM.

**Supplementary Table 7. Body weight change rate of PDX mice after drug treatment (%)**

| **Treatment** | **D0** | **D3** | **D6** | **D9** | **D12** |
| --- | --- | --- | --- | --- | --- |
| Vehicle | 0.0 ± 0.0 | -1.87 ± 1.57 | -1.11 ± 1.89 | 2.56 ± 1.84 | 5.40 ± 1.81 |
| THY-10A62  15 mg/kg | 0.0 ± 0.0 | -4.45 ± 0.82 | -2.52 ± 0.62 | -0.99 ± 1.08 | 4.16 ± 1.21 |
| THY-10A62  5 mg/kg | 0.0 ± 0.0 | -2.59 ± 0.43 | -0.87 ± 0.59 | 0.63 ± 0.96 | 3.61 ± 0.97 |
| PF-562271  15 mg/kg | 0.0 ± 0.0 | -1.61 ± 0.85 | 0.43 ± 1.92 | 1.85 ± 1.66 | 3.06 ± 1.32 |

**Note:** The day of group administration is D0. Data are presented as mean ± SEM.

**Supplementary Table 8. Statistics of tumor volume of PDX mice after drug treatment (mm^3^)**

| **Treatment** | **D0** | **D3** | **D6** | **D9** | **D12** |
| --- | --- | --- | --- | --- | --- |
| Vehicle | 115.9±75.3 | 286.3±156.9 | 646.5±351.8 | 1,078.5±238.2 | 1,736.6±340.3 |
| THY-10A62 15 mg/kg | 96.8±18.4 | 161.1±29.7  (p = 0.060) | 376.2±104.3**^*^**  (p = 0.035) | 538.0±134.0^*^  (p = 0.042) | 946.9±219.9^*^  (p = 0.042) |
| THY-10A62 5 mg/kg | 116.1±60.9 | 173.2±53.7 | 410.7±220.8 | 710.4±175.5 | 1,390.2±328.9 |
| PF-562271 15 mg/kg | 115.4±53.1 | 246.4±123.8 | 531.2±299.3 | 891.7±206.3 | 1,364.5±280.5 |

**Note:** The day of group administration is D0. Data are presented as mean ± SEM. *p < 0.05 compared with the Vehicle group.

**Supplementary Table 9. The difference of protein phosphorylation expression profile between THY-10A62 (15mg/kg) group and Vehicle group**

| **Uniprot ID** | **Phosphorylation site** | **Foldchange**  **(THY v.s. Vehicle)** | **Protein Name** | **Foldchange**  **(THY v.s. Vehicle)** |
| --- | --- | --- | --- | --- |
| **P31750** | **/** | **/** | **AKT1** | **0.72** |
| **P63260** | **/** | **/** | **ACTG1** | **0.82** |
| O35099 | ASK1 (Phospho-Ser966) | 1.90 | MAP3K5 | 1.22 |
| **Q61337** | **BAD (Phospho-Ser136)** | **1.39** | **BAD** | **/** |
| Q64373 | BCL-XL (Phospho-Ser62) | 2.28 | BCL2L1 | 1.70 |
| **P28028** | **B-RAF (Phospho-Ser446)** | **0.80** | **BRAF** | **0.67** |
| Q8C3Q9 | Caspase 9 (Phospho-Ser196) | 0.79 | CASP9 | 0.69 |
| P11440 | CDK1/CDC2 (Phospho-Tyr15) | 1.86 | CDK1 | 1.46 |
| Q64010 | CrkII (Phospho-Tyr221) | 1.26 | CRK | 1.20 |
| **P34152** | **FAK (Phospho-Ser910)** | **0.83** | **PTK2** | **0.73** |
| P16092 | FGFR1 (Phospho-Tyr654) | 0.66 | FGFR1 | 0.61 |
| Q9WVH4 | FKHRL1/FOXO3A (Phospho-Ser253) | 1.49 | FOXO3 | 1.58 |
| Q9QYY0 | Gab1 (Phospho-Tyr627) | 0.78 | GAB1 | 0.89 |
| Q99MK8 | GRK2 (Phospho-Ser29) | 0.81 | GRK2 | 0.71 |
| P11499 | HSP90B (Phospho-Ser226) | 4.39 | HSP90AB1 | 1.46 |
| O54910 | IkB-epsilon (Phospho-Ser22) | 1.22 | NFKBIE | 1.43 |
| O54890 | Integrin beta-3 (Phospho-Tyr773) | 1.49 | ITGB3 | 1.63 |
| P15261 | Interferon-gamma receptor alpha (Phospho-Tyr457) | 0.74 | IFNAR1 | 0.81 |
| Q62120 | JAK2 (Phospho-Tyr1007) | 1.35 | JAK2 | 1.26 |
| Q62120 | JAK2 (Phospho-Tyr221) | 1.57 | JAK2 | 1.26 |
| P06240 | LCK (Phospho-Tyr393) | 1.30 | LCK | 1.32 |
| Q08874 | MITF (Phospho-Ser73) | 0.79 | MITF | 0.69 |
| Q8C050 | MSK1 (Phospho-Ser376) | 1.40 | RPS6KA5 | 1.31 |
| Q04207 | NFkB-p65 (Phospho-Ser311) | 1.67 | RELA | 1.28 |
| Q8BSK8 | P70S6K (Phospho-Ser424) | 1.60 | RPS6KB1 | 2.16 |
| **P27671** | **Ras-GRF1 (Phospho-Ser916)** | **0.89** | **RASGRF1** | **0.70** |
| P13405 | Rb (Phospho-Ser608) | 1.20 | RB1 | 2.08 |
| Q04863 | RelB (Phospho-Ser552) | 1.41 | RELB | 1.28 |
| P98083 | Shc (Phospho-Tyr349) | 1.62 | SHC1 | 1.47 |
| Q62432 | Smad2 (Phospho-Ser467) | 0.87 | SMAD2 | 0.59 |
| Q62432 | Smad2 (Phospho-Thr8) | 0.79 | SMAD2 | 0.59 |
| Q8BUN5 | Smad3 (Phospho-Thr8) | 0.79 | SMAD3 | 0.78 |
| P52633 | STAT6 (Phospho-Thr645) | 0.90 | STAT6 | 0.75 |
| O55042 | Synuclein alpha (Phospho-Tyr136) | 1.64 | SNCA | 1.28 |
| P15209 | Trk B (Phospho-Tyr515) | 1.23 | NTRK2 | 1.55 |
| P70460 | VASP (Phospho-Ser157) | 1.68 | VASP | 1.58 |
| P35918 | VEGFR2 (Phospho-Tyr1175) | 1.57 | KDR | 1.22 |
| P35918 | VEGFR2 (Phospho-Tyr1214) | 1.34 | KDR | 1.22 |
| P43404 | Zap-70 (Phospho-Tyr319) | 2.34 | ZAP70 | 1.51 |

**Note:** Foldchange >1.20 or foldchange <0.83 were considered as differential expression.

**Supplementary Table 10. Quantitative PCR analysis of RNA expression levels of FAK (PTK2) and related downstream genes in tumor tissues**

|  | **Vehicle** | | | **THY-10A62 15 mg/kg** | | | **P value** |
| --- | --- | --- | --- | --- | --- | --- | --- |
|  | **Mean** | **SEM** | **Count** | **Mean** | **SEM** | **Count** |  |
| **PTK2** | 1.000 | 0.053 | 3 | 0.308 | 0.086 | 3 | 0.000 |
| **BRAF** | 1.000 | 0.041 | 3 | 0.498 | 0.246 | 3 | 0.025 |
| **BAD** | 1.000 | 0.036 | 3 | 1.040 | 0.073 | 3 | 0.443 |
| **AKT1** | 1.000 | 0.060 | 3 | 0.870 | 0.073 | 3 | 0.076 |
| **RASGRF1** | 1.000 | 0.097 | 3 | 0.679 | 0.101 | 3 | 0.017 |
| **Actin** | 1.000 | 0.050 | 3 | 0.880 | 0.033 | 3 | 0.026 |
